# Supplementary material for: DddA homolog search and engineering expand sequence compatibility of mitochondrial base editing
Source: Nat Commun. 2023 Feb 16;14:874. doi: 10.1038/s41467-023-36600-2 (PMC9935910; doi:10.1038/s41467-023-36600-2)

Supplementary Information

DddA homolog search and engineering expand sequence compatibility of mitochondrial base editing

Mi et al.

*To whom correspondence should be addressed to. Tel: +8610-62766945; Fax: +8610-62766945;

Email: yangming.wang@pku.edu.cn (YW)

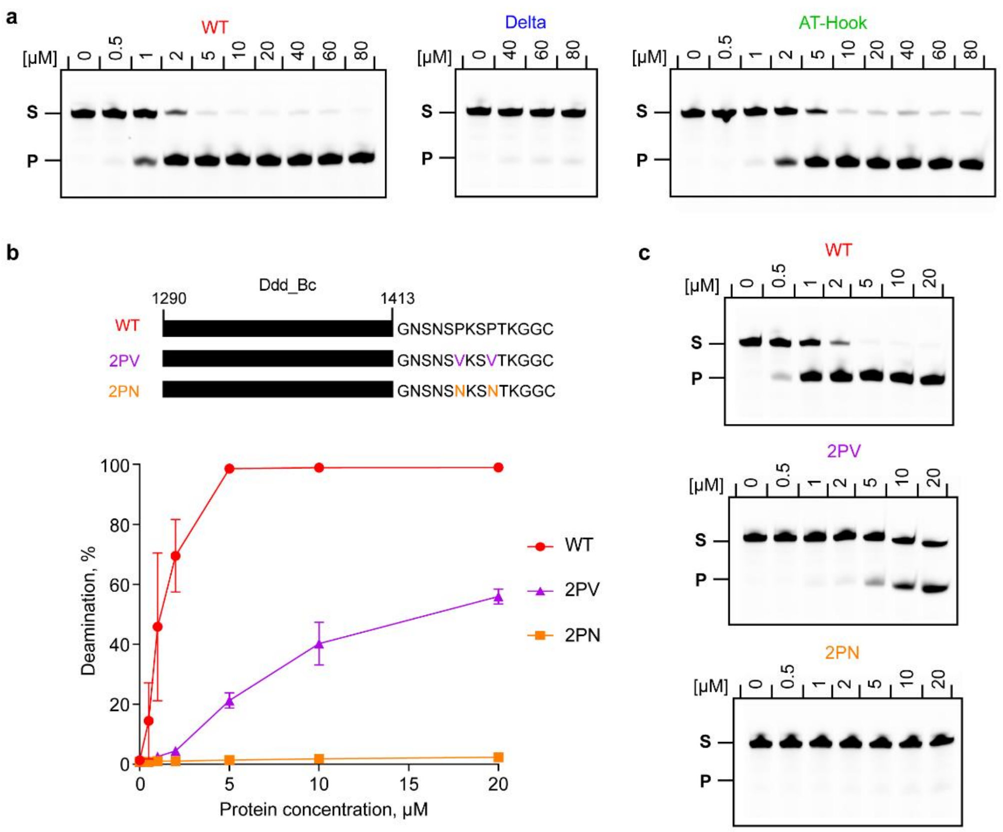

Supplementary Fig. 1 | SPKK-related motifs are important for the deamination activity of Ddd_Bc. a, In vitro cytidine deamination assays by wild type Ddd_Bc (WT), Ddd_Bc with two SPKK-related motifs removed (Delta), and Delta rescued with AT- hook sequence (AT-Hook) on 6-Carboxyfluorescein (FAM) labelled dsDNA substrate (S). The DNA sequence is 5'-FAM-ATATTATTTATTTTCATTTTATTTATTATA-3'. Cytidine deamination leads to products (P) with increased mobility. The samples derive from the same experiment and that gels were processed in parallel. Gels are representatives from n = 3 independent experiments. **b**, Quantification of the relative amounts of deamination product versus protein concentration for Ddd_Bc (WT) and two Ddd_Bc variants (2PV, 2PN), top shows the schematic of constructs. Associate gels are shown in **c**. Shown are mean ± SD; n = 3 independent experiments. **c**, In vitro cytidine deamination assays by wild type Ddd_Bc (WT) and two Ddd_Bc variants (2PV, 2PN) on 6-FAM labelled dsDNA. The sequence of dsDNA substrate is the same as shown in **a**. The samples derive from the same experiment and gels were processed in parallel. Gels are representatives from n = 3 independent experiments. Source data are provided as a Source Data file.

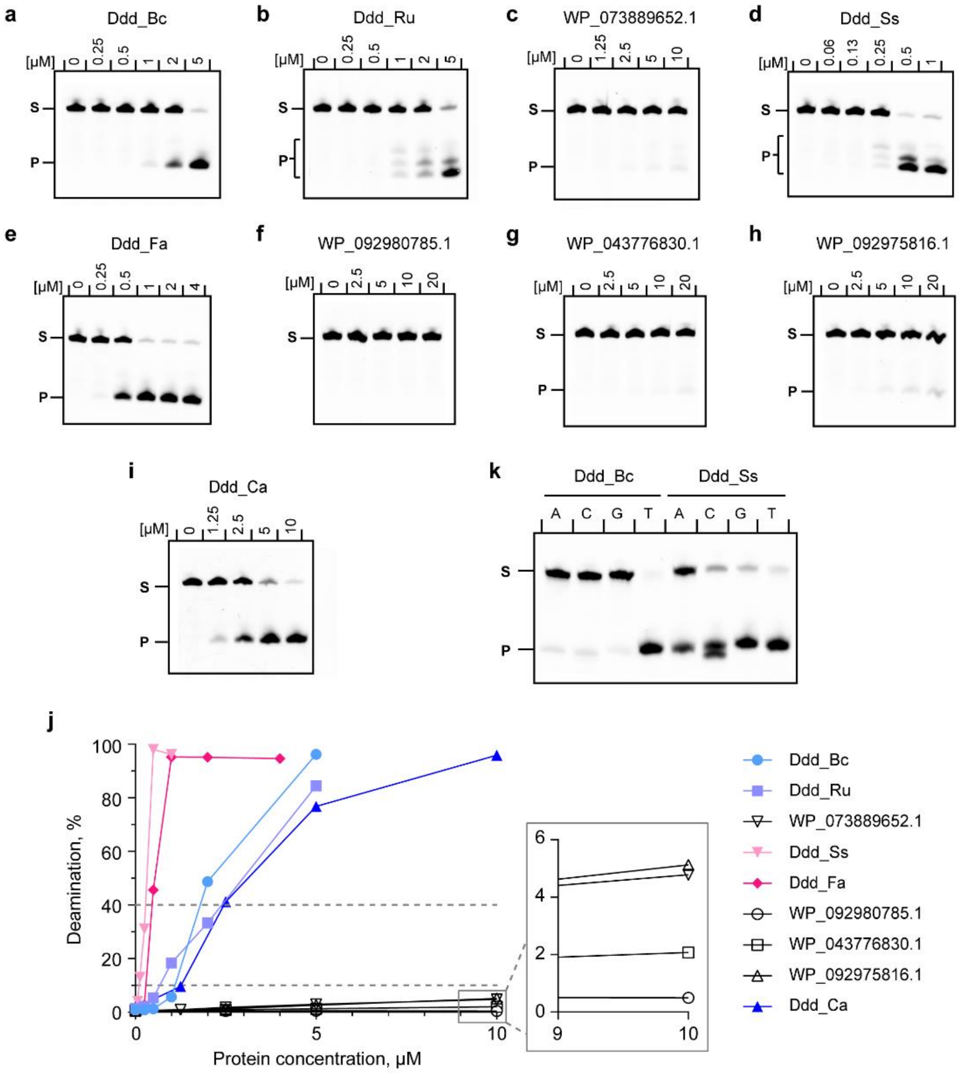

Supplementary Fig. 2 | Verification of deaminase activity for candidate Ddd_Bc homologs. a-i, In vitro cytidine deamination assays using a double-stranded 5'-FAM-ATATTTTCACGCCATTTATTA-3' DNA substrate, which contains AC, TC, CC and GC, with Ddd_Bc (**a**), Ddd_Ru (**b**), WP_073889652.1 (**c**), Ddd_Ss (**d**), Ddd_Fa (**e**), WP_092980785.1 (**f**), WP_043776830.1 (**g**), WP_092975816.1 (**h**) and Ddd_Ca (**i**). The samples derive from the same experiment and gels were processed in parallel. Gels are representatives of n = 2 independent experiments. **j**, Quantification of the relative amounts of deamination product versus protein concentration for Ddd_Bc and candidate Ddd_Bc homologs, associated gels are shown in **a-i**. Shown are mean from n = 2 independent experiments. **k**, In vitro cytidine deamination assays by Ddd_Bc and Ddd_Ss using 6-FAM labeled dsDNA substrate. The DNA sequence is 5'-FAM-ATATTATTTGNCATTTATTATA-3', and the N is indicated at the top of each lane. Shown is a

representative gel from n = 3 independent experiments. Source data are provided as a Source Data file.

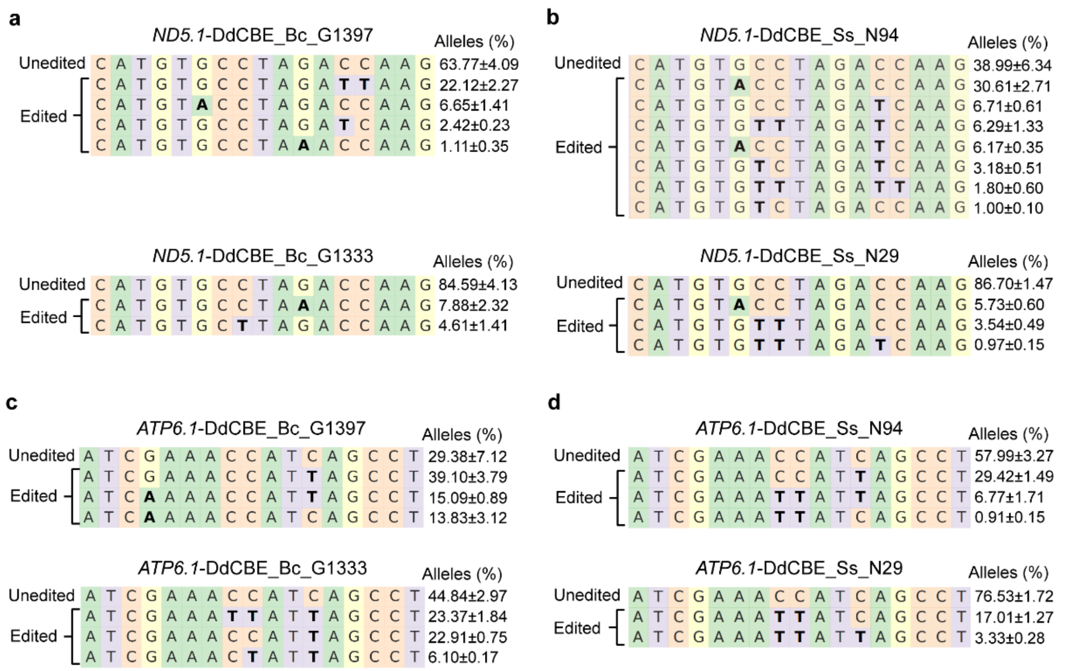

Supplementary Fig. 3 | Allele compositions from mitochondrial editing by ND5.1-DdCBEs and ATP6.1-DdCBEs. a, **b**, Frequencies of *MT-ND5* alleles produced by *ND5.1*-DdCBE_Bc (**a**) with G1397 and G1333 split or *ND5.1*-DdCBE_Ss (**b**) with N94 and N29 split. **c**, **d**, Frequencies of *MT-ATP6* alleles produced by *ATP6.1*-DdCBE_Bc (**c**) with G1397 and G1333 split or *ATP6.1*-DdCBE_Ss (**d**) with N94 and N29 split. Shown are mean ± SD; n = 3 independent experiments.

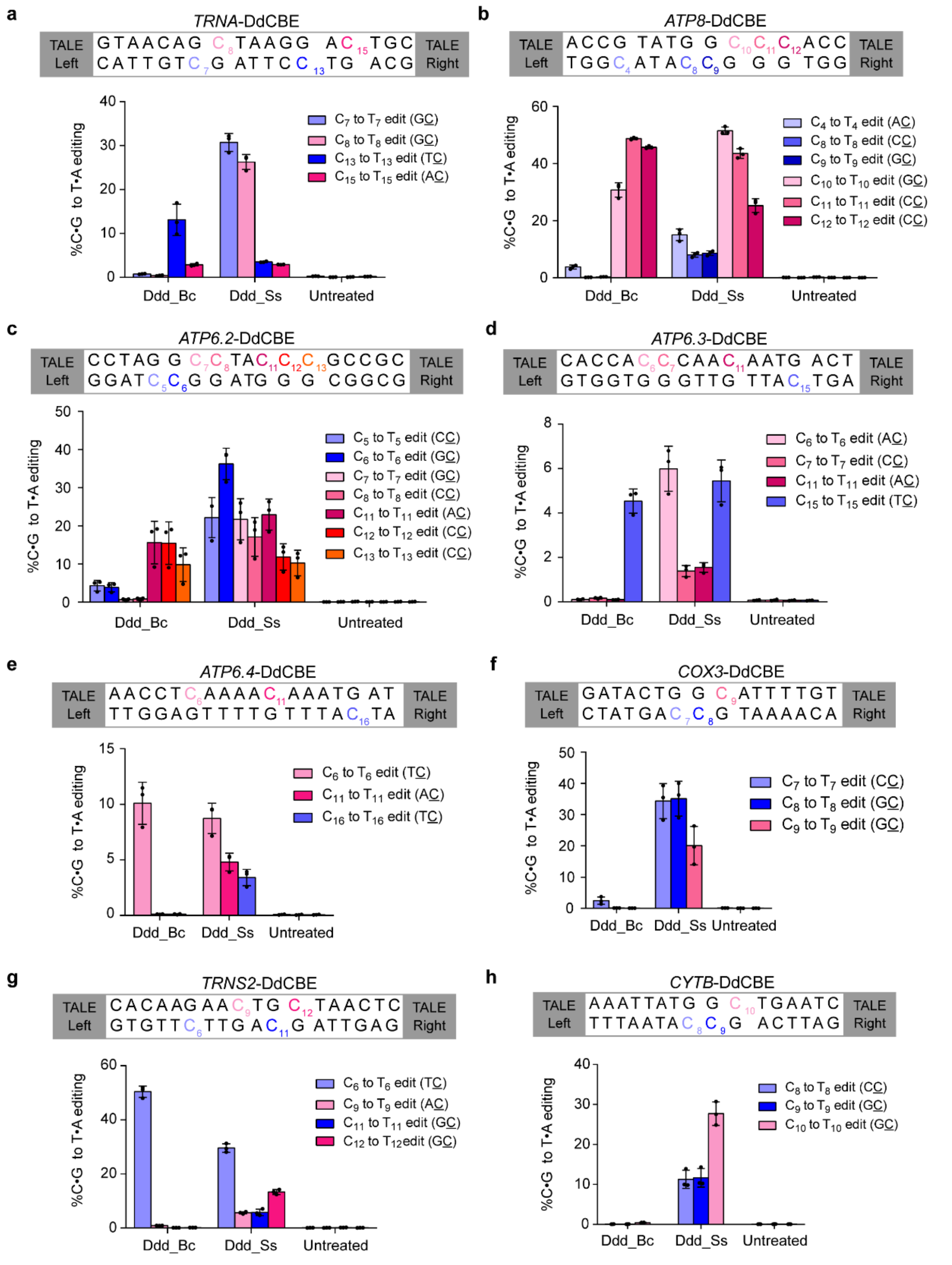

Supplementary Fig. 4 | Ddd_Ss enable efficient editing at 8 mitochondrial DNA sites. a-h, mtDNA editing efficiencies of HEK293T cells treated with *TRNA*-DdCBE (**a**), *ATP8*-DdCBE (**b**), *ATP6.2*-DdCBE (**c**), *ATP6.3*-DdCBE (**d**), *ATP6.4*-DdCBE (**e**), *COX3*-DdCBE (**f**), *TRNS2*-DdCBE (**g**), *CYTB*-DdCBE (**h**). Shown are mean ± SD; n = 3 independent experiments. Source data are provided as a Source Data file.

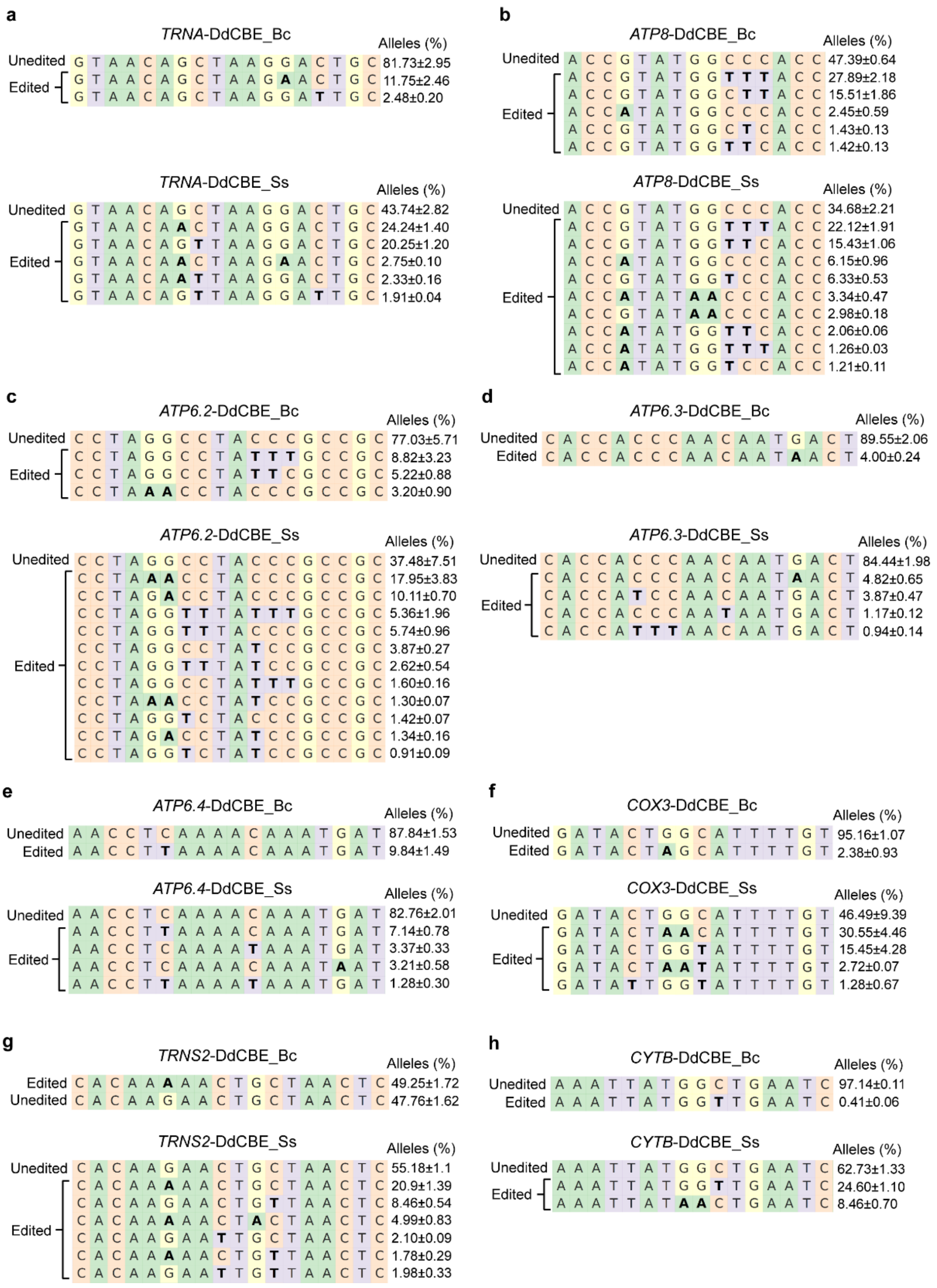

Supplementary Fig. 5 | Allele compositions for 8 mitochondrial DNA sites edited by DdCBE_Bc or DdCBE_Ss. a-h, Frequencies of DdCBE edited alleles produced by *TRNA*-DdCBE (**a**), *ATP8*-DdCBE (**b**), *ATP6.2*-DdCBE (**c**), *ATP6.3*-DdCBE (**d**), *ATP6.4*-DdCBE (**e**), *COX3*-DdCBE (**f**), *TRNS2*-DdCBE (**g**), *CYTB*-DdCBE (**h**). Shown are mean ± SD; n = 3 independent experiments.

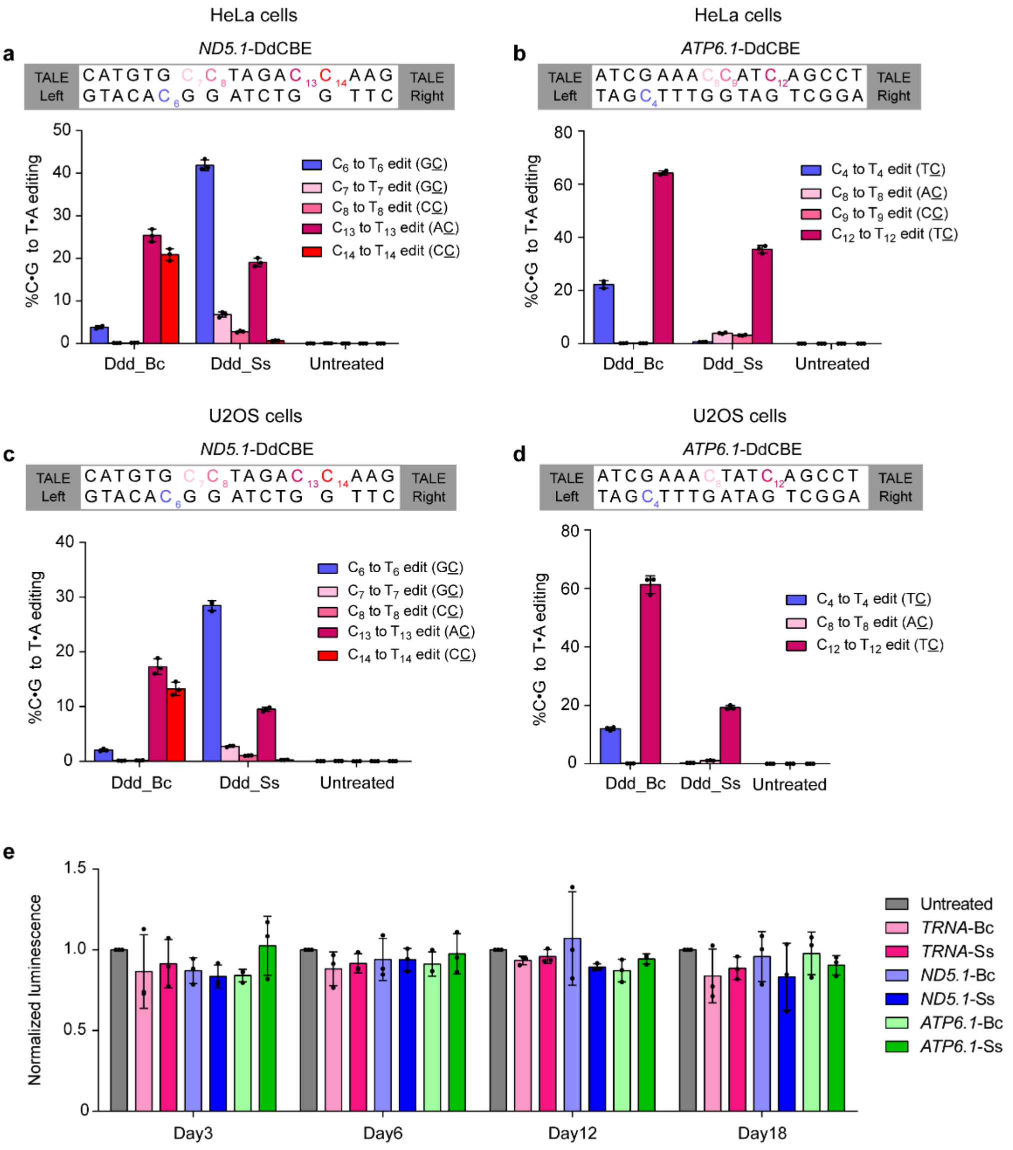

Supplementary Fig. 6 | DdCBE_Ss can enable mitochondrial base editing in other human cell lines. a, **b**, mtDNA editing efficiencies of HeLa cells treated with *ND5.1*-DdCBE (**a**) and *ATP6.1*-DdCBE (**b**). **c**, **d**, mtDNA editing efficiencies of U2OS cells treated with *ND5.1*-DdCBE (**c**) and *ATP6.1*-DdCBE (**d**). Shown are mean ± SD; n = 3 independent experiments. **e**, Cell viability was measured by recording the luminescence in HEK293T cells at various days after treated with DdCBE_Bc or DdCBE_Ss targeting different mtDNA loci. The values were normalized to the untreated samples from the same day. Shown are mean ± SD; n = 3 independent experiments. Source data are provided as a Source Data file.

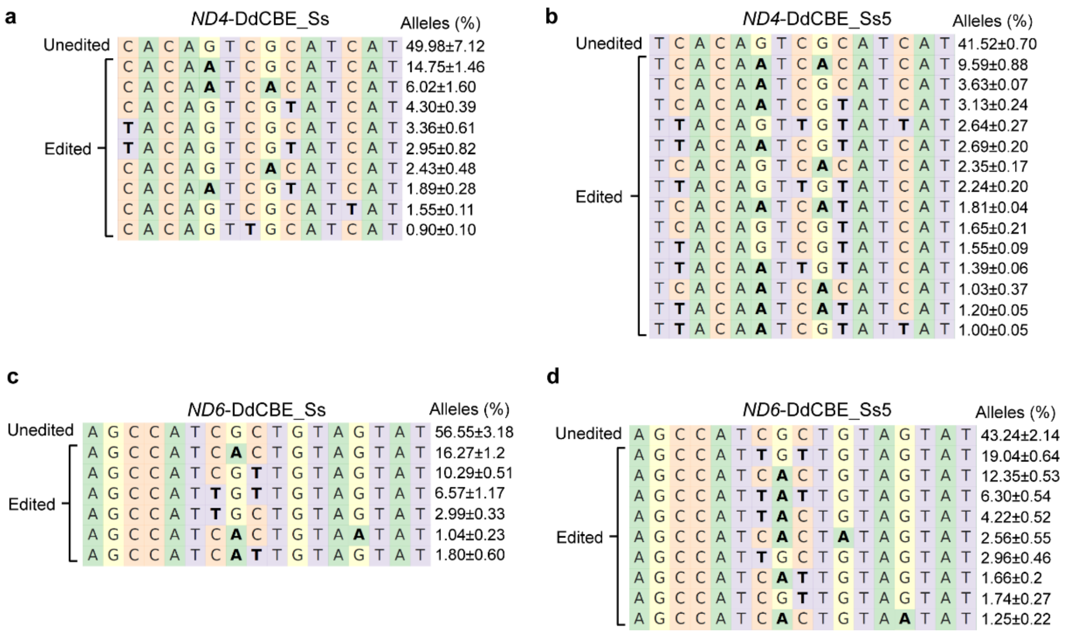

Supplementary Fig. 7 | Allele compositions from mitochondrial editing by ND4-DdCBEs and ND6-DdCBEs. a, **b**, Frequencies of *MT-ND4* alleles produced by *ND4*-DdCBE_Ss (**a**) or *ND4*-DdCBE_Ss5 (**b**). **c**, **d**, Frequencies of *MT-ND6* alleles produced by *ND6*-DdCBE_Ss (**c**) or *ND6*-DdCBE_Ss5 (**d**). Shown are mean ± SD. n = 3 independent experiments.

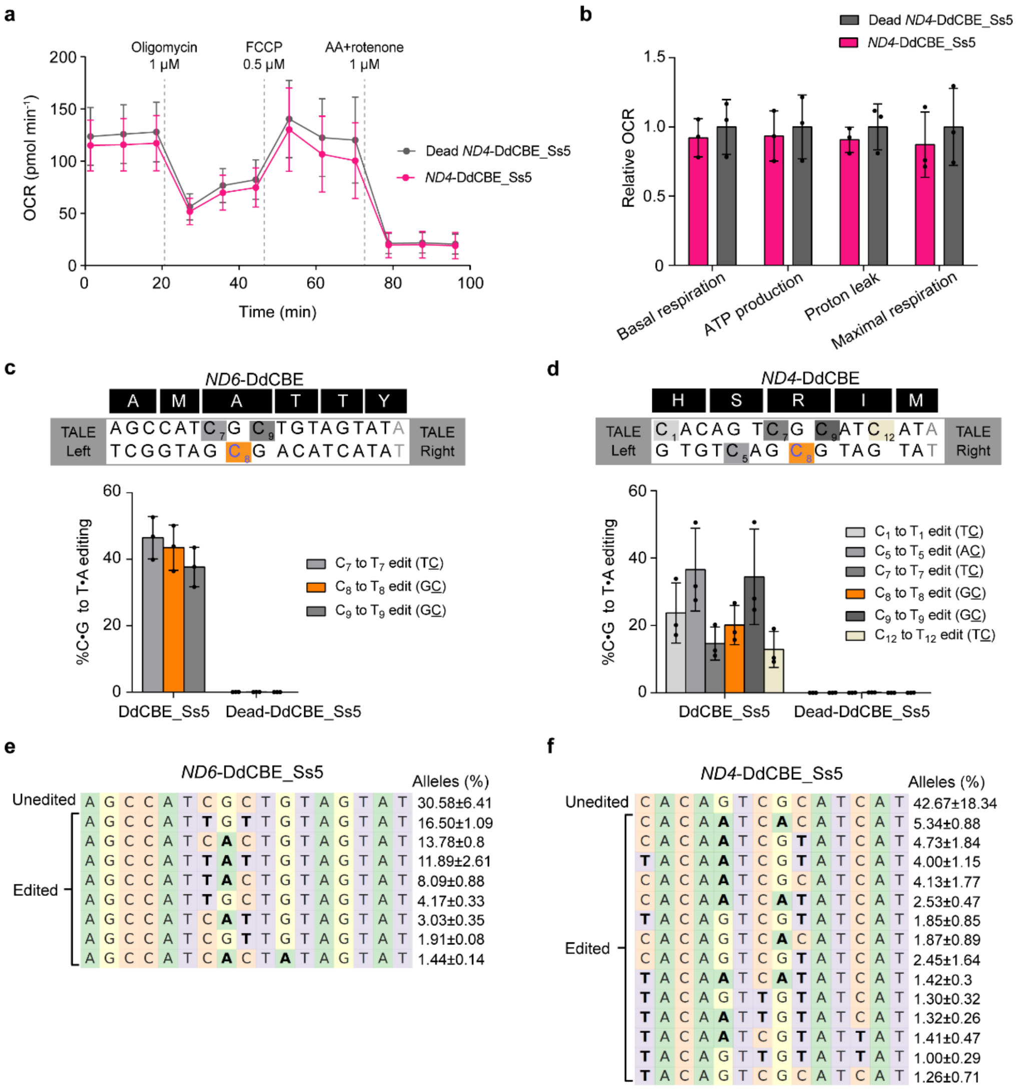

Supplementary Fig. 8 | DdCBE_Ss can install disease-associated mtDNA mutations in previously inaccessible GC sites. a, **b**, Oxygen consumption rate (OCR) (**a**) and relative values of respiratory parameters (**b**) in HEK293T cells treated with the *ND4*-DdCBE_Ss5 or dead *ND4*-DdCBE_Ss5. Shown are mean ± SD; n = 3 independent experiments. **c**, **d**, Mitochondrial base editing efficiencies of cells used in **Fig. 3d** and **Fig. 3e** (**c**), and cells used in **a** and **b** (**d**). Disease-associated target sites are shown using orange. Shown are mean ± SD; n = 3 independent experiments. **e**, **f**, Frequencies of *MT-ND6* (**e**) and *MT-ND4* (**f**) alleles produced by *ND6*- and *ND4*-DdCBE_Ss5 in cells used for OCR measurement. Shown are mean ± SD. n = 3 independent experiments. Source data are provided as a Source Data file.

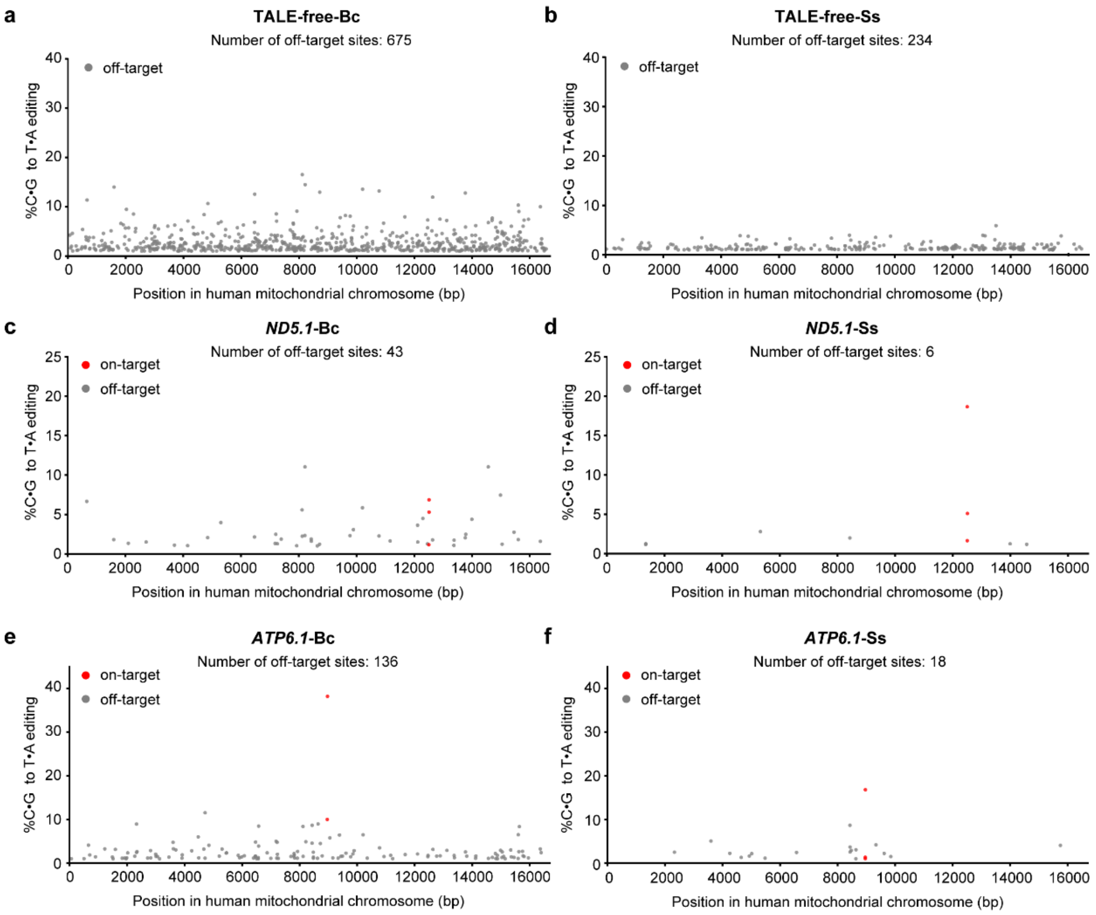

Supplementary Fig. 9 | The C•G to T•A editing frequency in mitochondrial genome of HEK293T cells treated with different DdCBEs. a-f, Average C•G to T•A editing efficiency of on-target (red dots) and off-target (gray dots) sites across mtDNA are shown for HEK293T cells treated with (**a**) DdCBE_Bc without TALE arrays, (**b**) DdCBE_Ss without TALE arrays, (**c**) *ND5.1*-DdCBE_Bc, (**d**) *ND5.1*-DdCBE_Ss, (**e**) *ATP6.1*-DdCBE_Bc, and (**f**) *ATP6.1*-DdCBE_Ss. Sites with average editing frequency greater than 1% are shown. Data are shown as means from n = 2 independent experiments. Source data are provided as a Source Data file.

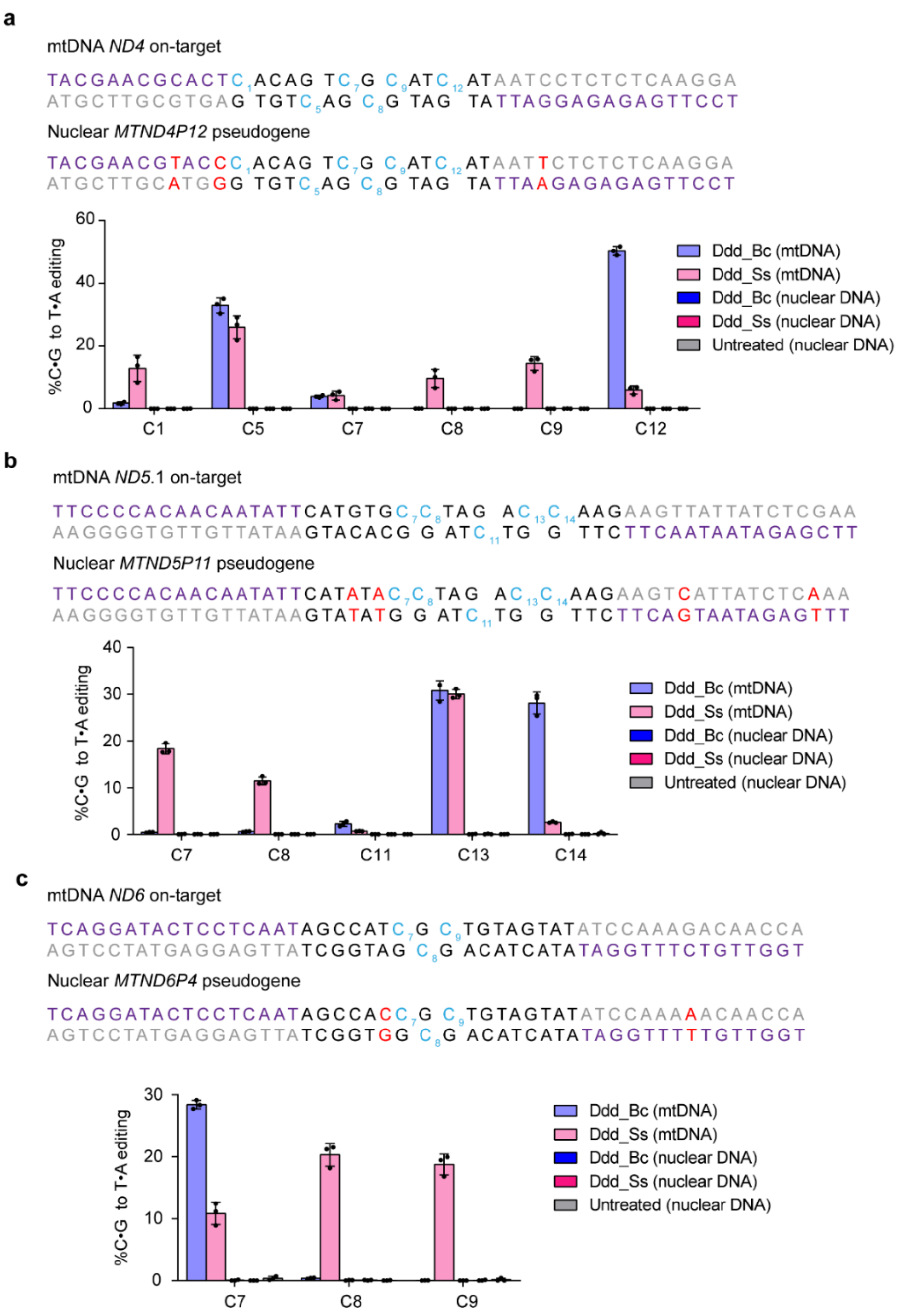

Supplementary Fig. 10 | Off-target editing activity of DdCBEs in nuclear genome of HEK293T cells. a-c, The on-target editing site in mtDNA and the corresponding sites in nuclear DNA with the greatest homology are shown for *ND4*-DdCBE (**a**), *ND5.1*-DdCBE (**b**) and *ND6*-DdCBE (**c**). TALE binding sites are shown in purple. Target cytosines are in blue. Nucleotide mismatches between the mtDNA and nuclear pseudogenes are in red. Shown are mean ± SD; n = 3 independent experiments. Source data are provided as a Source Data file.

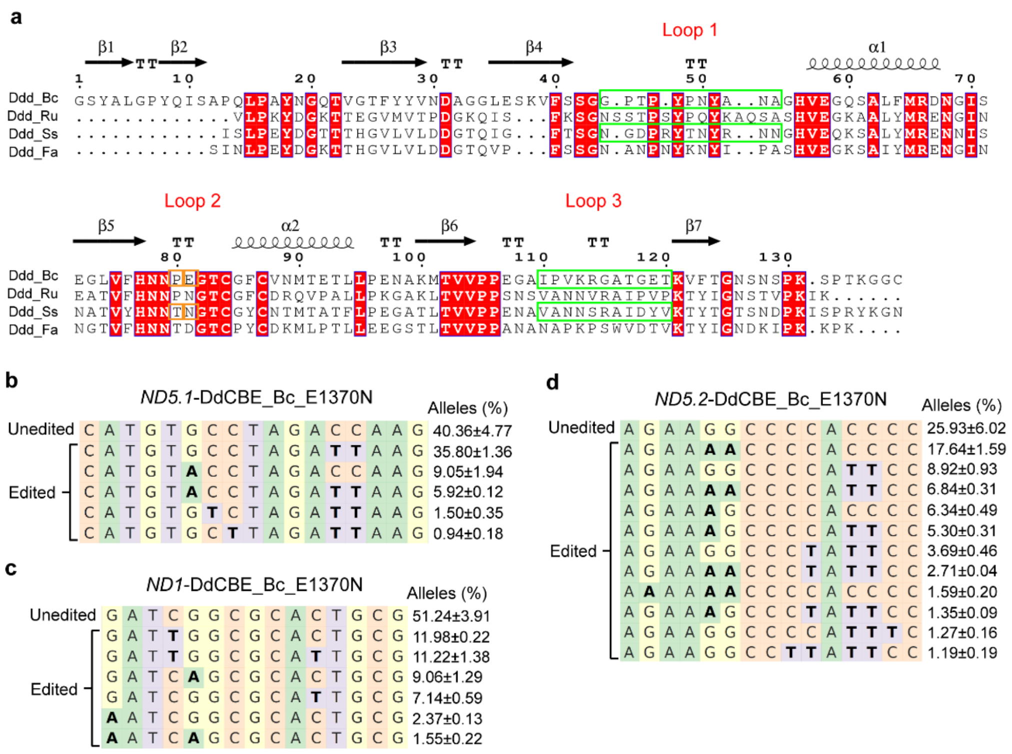

Supplementary Fig. 11 | E1370N mutation extensively increases the editing efficiency and sequence compatibility of DdCBE_Bc. **a**, Multiple alignment of Ddd_Bc, Ddd_Ru, Ddd_Ss and Ddd_Fa. Secondary structure elements are presented on top according to Ddd_Bc structure; helices with squiggles, β-strands with arrows, and turns with TT letters. Loop numbers are manually designated. Two orange boxes mark two amino acids P1369 and E1370 in Ddd_Bc and corresponding amino acids in Ddd_Ss. Green boxes mark the sequences that are used to make Ddd_Bc_L1 and Ddd_Bc_L3 variants. **b-d**, Frequencies of DdCBE edited alleles produced by *ND5.1*-DdCBE_Bc_E1370N (**b**), *ND1*-DdCBE_Bc_E1370N (**c**), *ND5.2*-DdCBE_Bc_E1370N (**d**). Shown are mean ± SD; n = 3 independent experiments.

Supplementary Table 1 | Primers for HTS of on-target sites targeted by DdCBE.

Site	HTS forward primer	HTS reverse primer
ND1	CCTACACGACGCTCTTCCGATCTNNNNNNAGCCTAGCCGTTTACTCAATCCTCTG	GTTCAGACGTGTGCTCTTCCGATCTGGGTGGAGAGGTTAAAGGAGCCAC
TRNA	CCTACACGACGCTCTTCCGATCTNNNNNNGGTTAAATACAGACCAAGAGCCTTCAAAGCC	GTTCAGACGTGTGCTCTTCCGATCTGGCTTAGCTTAATTAAAGTGGCTGATTTGCG
ATP8	CCTACACGACGCTCTTCCGATCTNNNNNNCTAGAGCCCACTGTAAAGCTAACTTAGC	GTTCAGACGTGTGCTCTTCCGATCTCTTTGGTGAGGGAGGTAGGTGGTAG
ATP6.1	CCTACACGACGCTCTTCCGATCTNNNNNNGCCCTAGCCCACTTCTTACCACAAG	GTTCAGACGTGTGCTCTTCCGATCTGGCCTGCAGTAATGTTAGCGGTTAGG
ATP6.2	CCTACACGACGCTCTTCCGATCTNNNNNNCCCTGAGAACCAAAATGAACGAAAATCTG	GTTCAGACGTGTGCTCTTCCGATCTGGGTGGTGATTAGTCGGTTGTTGATG
ATP6.3/ ATP6.4	CCTACACGACGCTCTTCCGATCTNNNNNNCTATTTCCCCCTCTATTGATCCCCAC	GTTCAGACGTGTGCTCTTCCGATCTGTCCGAGGAGGTTAGTTGTGGC
COX3	CCTACACGACGCTCTTCCGATCTNNNNNNCCGCCAACTAATATTTCACTTTACATCCAAACATCAC	GTTCAGACGTGTGCTCTTCCGATCTGAGTAAGACCCTCATCAATAGATGGAGACATACAG
ND4	CCTACACGACGCTCTTCCGATCTNNNNNNCATAATCGCCCACGGGCTTACATCC	GTTCAGACGTGTGCTCTTCCGATCTGCTAGAAGTCATCAAAAAGCTATTAGTGGGAGTAGAG
TRNS2	CCTACACGACGCTCTTCCGATCTNNNNNNGTGAATCTGACAACAGAGGCTTACGACC	GTTCAGACGTGTGCTCTTCCGATCTGGCCTAAGACCAATGGATAGCTGTTATCC
ND5.1	CCTACACGACGCTCTTCCGATCTNNNNNNCCATTGTCGCATCCACCTTTATTATCAGTCTC	GTTCAGACGTGTGCTCTTCCGATCTGTAGTCTAGTTTGAAGCTTAGGGAGAGCTGG
ND5.2	CCTACACGACGCTCTTCCGATCTNNNNNNGCAGCAGGCAAATCAGCCCAATTAG	GTTCAGACGTGTGCTCTTCCGATCTGGAAGCGGATGAGTAAGAAGATTCCTGC
ND6	CCTACACGACGCTCTTCCGATCTNNNNNNCTCACCAAGACCTCAACCCCTGAC	GTTCAGACGTGTGCTCTTCCGATCTCTGAATTTTGGGGGAGGTTATATGGGTTTAATAG
CYTB	CCTACACGACGCTCTTCCGATCTNNNNNNTGCACTACTCACCAGACGCCTCAAC	GTTCAGACGTGTGCTCTTCCGATCTCCCGATGTGTAGGAAGAGGCAG

Supplementary Table 2 | Primers for HTS of off-target sites in pseudogens.

Name	Forward primer	Reverse primer
Pseudogene MTND4P12/5P11/6P4	CTAATTCTCTTTGAGGAGCATGGTTAG	TATCACTTCCAGCCACCTATTTCC
MTND4P12-HTS	CCTACACGACGCTCTTCCGATCTNNNNNNGTCGCCCACGGACTTACATCCTCATTAC	GTTCAGACGTGTGCTCTTCCGATCTGGCGAGATTAGTGAGGCTTGCTAGAAGTC
MTND5P11-HTS	CCTACACGACGCTCTTCCGATCTNNNNNNCCCATTAAGTAAAATCCATTATCGCATCCACC	GTTCAGACGTGTGCTCTTCCGATCTGTTTGAAGCTTAGGGAGAGTTGGGTTG
MTND6P4-HTS	CCTACACGACGCTCTTCCGATCTNNNNNNCACTTACCAAGACCTCAACCCCTGACCC	GTTCAGACGTGTGCTCTTCCGATCTTTCTGAATTACGGGGGAGGTTACATGGG

Supplementary Note 1 | Sequences used to construct DdCBE architecture, *ND5.1*-DdCBE and TALE amino acid sequences.

TXN2 MTS

MAQRLLLRRFLASVISRKPSQGQWPPLTSRALQTPQCSPGGLTVTPNPARTIYTTRISLT

HA

YPYDVPDYA

FLAG

DYKDDDDK

N-Ddd_Bc_G1397

GSYALGPYQISAPQLPAYNGQTVGTFYYVNDAGGLESKVFSSGGPTPYPNYANAGHVE
GQSALFMRDNGISEGLVFHNNPEGTCGFCVNMTETLLPENAKMTVVPPEG

C-Ddd_Bc_G1397

AIPVKRGATGETKVFTGNSNSPKSPTKGGC

N-Ddd_Ss_N94

SLPEYDGTTTHGVLVLDDGTQIGFTSGNGDPRYTNYRNNGHVEQKSALYMRENNISNAT
VYHNNTNGTCGYCNTMTATFLPEGATLTVVPPEN

C-Ddd_Ss_N94

AVANNSRAIDYVKTYTGTSNDPKISPRYKGN

N-Ddd_Bc_G1333

GSYALGPYQISAPQLPAYNGQTVGTFYYVNDAGGLESKVFSSGG

C-Ddd_Bc_G1333

PTPYPNYANAGHVEGQSALFMRDNGISEGLVFHNNPEGTCGFCVNMTETLLPENAKMT
VVPPEGAIPVKRGATGETKVFTGNSNSPKSPTKGGC

N-Ddd_Ss_N29

SLPEYDGTTTHGVLVLDDGTQIGFTSGN

C-Ddd_Ss_N29

GDPRYTNYRNNGHVEQKSALYMRENNISNATVYHNNTNGTCGYCNTMTATFLPEGATL
TVVPPENAVANNSRAIDYVKTYTGTSNDPKISPRYKGN

UGI

TNLSDIIEKETGKQLVIQESILMLPEEVEEVIGNKPESDILVHTAYDESTDENVMLLTSDAPE
YKPWALVIQDSNGENKIKML

T2A

GSGEGRGSLLTCGDVEENPGP

GFP

MVSKGEELFTGVVPILVELDGDVNGHKFSVSGEGEGDATYGKLTLKFICTTGKLPVPWP
TLVTTLTYGVQCFSRYPDHMKQHDFFKSAMPEGYVQERTIFFKDDGNYKTRAEVKFEG
DTLVNRIELKGIDFKEDGNILGHKLEYNYNSHNVYIMADKQKNGIKVNFKIRHNIEDGSVQ
LADHYQQNTPIGDGPVLLPDNHYLSTQSALSKDPNEKRDHMVLLEFVTAAGITLGMDEL
YK

mCherry

MVSKGEEDNMAIIKEFMRFKVHMEGSVNGHEFEIEGEGEGRPYEGTQTAKLKVTKGGP

LPFAWDILSPQFMYGSKAYVKHPADIPDYLKLSFPEGFKWERVMNFEDGGVVTVTQDSS
LQDGEFIYKVKLRGTNFPSDGPVMQKKTMGWEASSERMYPEDGALKGEIKQRLKLKDG
GHYDAEVKTTYKAKKPVQLPGAYNVNIKLDITSHNEDYTIVEQYERAEGRHSTGGMDEL
YK

2aa linker

GS

4aa linker

SGGS

The general architecture of left-side halves of DdCBEs (from N- to C-terminus):

TXN2 MTS-HA-TALE-2aa linker-Ddd_Bc/Ddd_Ss half-4aa linker-UGI-T2A-GFP

The general architecture of right-side halves of DdCBEs (from N- to C-terminus):

TXN2 MTS-FLAG-TALE-2aa linker-Ddd_Bc/Ddd_Ss half-4aa linker-UGI-T2A-GFP

ND5.1-DdCBE_Bc_G1397-Left:

MAQRLLLRRFLASVISRKPSQGQWPPLTSRALQTPQCSPGGLTVTPNPARTIYTTRISLT
YPYDVPDYASGAS**VDLRTLGYSQQQQEKIKPKVRSTVAQHHEALVGHGFTHAHIVALS
QHPAALGTVAVKYQDMIAALPEATHEAIVGVGKQWSGARALEALLTVAGELRGPPLQL
DTGQLLKIAKRGGVTAVEAVHAWRNALTGAPLN**LTPEQVVAIASNGGGKQALETVQRLL
PVLCQAHGLTPEQVVAIASHDGGKQALETVQRLLPVLCQAHGLTPEQVVAIASHDGGKQ
ALETVQRLLPVLCQAHGLTPEQVVAIASHDGGKQALETVQRLLPVLCQAHGLTPEQVVAI
ASHDGGKQALETVQRLLPVLCQAHGLTPEQVVAIASNIGGKQALETVQRLLPVLCQAHG
LTPEQVVAIASHDGGKQALETVQRLLPVLCQAHGLTPEQVVAIASNIGGKQALETVQRLL
PVLCQAHGLTPEQVVAIASNIGGKQALETVQRLLPVLCQAHGLTPEQVVAIASHDGGKQA
LETVQRLLPVLCQAHGLTPEQVVAIASNIGGKQALETVQRLLPVLCQAHGLTPEQVVAIAS
NIGGKQALETVQRLLPVLCQAHGLTPEQVVAIASNGGGKQALETVQRLLPVLCQAHGLT
PEQVVAIASNIGGKQALETVQRLLPVLCQAHGLTPEQVVAIASNGGGKQALETVQRLLPV
LCQAHGLTPEQVVAIASNGGGKQALE**SIVAQLSRPDPALAALTNDHLVALACLGGRPAL
DAVKKGLG**GSGSYALGPYQISAPQLPAYNGQTVGTFYYVNDAGGLESKVFSSGGPTPY
PNYANAGHVEGQSALFMRDNGISEGLVFHNNPEGTCGFCVNMTETLLPENAKMTVVPP
EGSGGSTNLSDIIEKETGKQLVIQESILMLPEEVEEVIGNKPESDILVHTAYDESTDENVML
LTSDAPEYKPWALVIQDSNGENKIKMLSGVDGSGEGRGSLLTCGDVEENPGPPVATMVS
KGEELFTGVVPILVELDGDVNGHKFSVSGEGEGDATYGKLTLKFICTTGKLPVPWPTLVT
TLTYGVQCFSRYPDHMKQHDFFKSAMPEGYVQERTIFFKDDGNYKTRAEVKFEGDTLV
NRIELKGIDFKEDGNILGHKLEYNYNSHNVYIMADKQKNGIKVNFKIRHNIEDGSVQLADH
YQQNTPIGDGPVLLPDNHYLSTQSALSKDPNEKRDHMVLLEFVTAAGITLGMDELYKLE

ND5.1-DdCBE_Bc_G1397-Right:

MAQRLLLRRFLASVISRKPSQGQWPPLTSRALQTPQCSPGGLTVTPNPARTIYTTRISLT
DYKDDDDKSGAS**VDLRTLGYSQQQQEKIKPKVRSTVAQHHEALVGHGFTHAHIVALSQ
HPAALGTVAVKYQDMIAALPEATHEAIVGVGKQWSGARALEALLTVAGELRGPPLQLD
TGQLLKIAKRGGVTAVEAVHAWRNALTGAPLN**LTPEQVVAIASNGGGKQALETVQRLLP

VLCQAHGLTPEQVVAIASHDGGKQALETVQRLLPVLCQAHGLTPEQVVAIASNNGGKQA
LETVQRLLPVLCQAHGLTPEQVVAIASNIGGKQALETVQRLLPVLCQAHGLTPEQVVAIAS
NNGGKQALETVQRLLPVLCQAHGLTPEQVVAIASNIGGKQALETVQRLLPVLCQAHGLT
PEQVVAIASNGGGKQALETVQRLLPVLCQAHGLTPEQVVAIASNIGGKQALETVQRLLPV
LCQAHGLTPEQVVAIASNIGGKQALETVQRLLPVLCQAHGLTPEQVVAIASNGGGKQALE
TVQRLLPVLCQAHGLTPEQVVAIASNIGGKQALETVQRLLPVLCQAHGLTPEQVVAIASNI
GGKQALETVQRLLPVLCQAHGLTPEQVVAIASHDGGKQALETVQRLLPVLCQAHGLTPE
QVVAIASNGGGKQALETVQRLLPVLCQAHGLTPEQVVAIASNGGGKQALE**SIVAQLSRP**
DPALAALTNDHLVALACLGGRPALDAVKKGLGGSAIPVKRGATGETKVFTGNSNSPKS
PTKGGCSGGSTNLSDIIEKETGKQLVIQESILMLPEEVEEVIGNKPESDILVHTAYDESTDE
NVMLLTSDAPEYKPWALVIQDSNGENKIKMLSGVDGSGEGRGSLLTCGDVEENPGPPV
ATMVSKGEELFTGVVPILVELDGDVNGHKFSVSGEGEGDATYGKLTLKFICTTGKLPVPW
PTLVTTLTYGVQCFSRYPDHMKQHDFFKSAMPEGYVQERTIFFKDDGNYKTRAEVKFE
GDTLVNRIELKGIDFKEDGNILGHKLEYNYNSHNVYIMADKQKNGIKVNFKIRHNIEDGSV
QLADHYQQNTPIGDGPVLLPDNHYLSTQSALSKDPNEKRDHMVLLEFVTAAGITLGMDE
LYKLE

When construct DdCBE without T2A-GFP sequence, the underlined sequence was removed.

When construct DdCBE with T2A-mCherry sequence, the GFP sequence was replaced by mCherry sequence.

ND5.1-DdCBE_Ss_N94-Left:

MAQRLLLRRFLASVISRKPSQGQWPPLTSRALQTPQCSPGGLTVTPNPARTIYTTRISLT
YPYDVPDYASGAS**VDLRTLGYSQQQQEKIKPKVRSTVAQHHEALVGHGFTHAHIVALS**
QHPAALGTVAVKYQDMIAALPEATHEAIVGVGKQWSGARALEALLTVAGELRGPPLQL
DTGQLLKIAKRGGVTAVEAVHAWRNALTGAPLNLTPEQVVAIASNGGGKQALETVQRLL
PVLCQAHGLTPEQVVAIASHDGGKQALETVQRLLPVLCQAHGLTPEQVVAIASHDGGKQ
ALETVQRLLPVLCQAHGLTPEQVVAIASHDGGKQALETVQRLLPVLCQAHGLTPEQVVAI
ASHDGGKQALETVQRLLPVLCQAHGLTPEQVVAIASNIGGKQALETVQRLLPVLCQAHG
LTPEQVVAIASHDGGKQALETVQRLLPVLCQAHGLTPEQVVAIASNIGGKQALETVQRLL
PVLCQAHGLTPEQVVAIASNIGGKQALETVQRLLPVLCQAHGLTPEQVVAIASHDGGKQA
LETVQRLLPVLCQAHGLTPEQVVAIASNIGGKQALETVQRLLPVLCQAHGLTPEQVVAIAS
NIGGKQALETVQRLLPVLCQAHGLTPEQVVAIASNGGGKQALETVQRLLPVLCQAHGLT
PEQVVAIASNIGGKQALETVQRLLPVLCQAHGLTPEQVVAIASNGGGKQALETVQRLLPV
LCQAHGLTPEQVVAIASNGGGKQALE**SIVAQLSRPDPALAALTNDHLVALACLGGRPAL**
DAVKKGLGGSSLPEYDGTTTHGVLVLDDGTQIGFTSGNGDPRYTNYRNNGHVEQKSAL
YMRENNISNATVYHNNTNGTCGYCNTMTATFLPEGATLTVVPPENSGGSTNLSDIIEKET
GKQLVIQESILMLPEEVEEVIGNKPESDILVHTAYDESTDENVMLLTSDAPEYKPWALVIQ
DSNGENKIKMLSGVDGSGEGRGSLLTCGDVEENPGPPVATMVSKGEELFTGVVPILVEL
DGDVNGHKFSVSGEGEGDATYGKLTLKFICTTGKLPVPWPTLVTTLTYGVQCFSRYPDH
MKQHDFFKSAMPEGYVQERTIFFKDDGNYKTRAEVKFEGDTLVNRIELKGIDFKEDGNIL
GHKLEYNYNSHNVYIMADKQKNGIKVNFKIRHNIEDGSVQLADHYQQNTPIGDGPVLLP

DNHYLSTQSALSKDPNEKRDHMVLLEFVTAAGITLGMDELYKLE

ND5.1-DdCBE_Ss_N94-Right:
MAQRLLLRRFLASVISRKPSQGQWPPLTSRALQTPQCSPGGLTVTPNPARTIYTTRISLT
DYKDDDDKSGAS**VDLRTLGYSQQQQEKIKPKVRSTVAQHHEALVGHGFTHAHIVALSQ**
HPAALGTVAVKYQDMIAALPEATHEAIVGVGKQWSGARALEALLTVAGELRGPPLQLD
TGQLLKIAKRGGVTAVEAVHAWRNALTGAPLNLTPEQVVAIASNGGGKQALETVQRLLP
VLCQAHGLTPEQVVAIASHDGGKQALETVQRLLPVLCQAHGLTPEQVVAIASNNGGKQA
LETVQRLLPVLCQAHGLTPEQVVAIASNIGGKQALETVQRLLPVLCQAHGLTPEQVVAIAS
NNGGKQALETVQRLLPVLCQAHGLTPEQVVAIASNIGGKQALETVQRLLPVLCQAHGLT
PEQVVAIASNGGGKQALETVQRLLPVLCQAHGLTPEQVVAIASNIGGKQALETVQRLLPV
LCQAHGLTPEQVVAIASNIGGKQALETVQRLLPVLCQAHGLTPEQVVAIASNGGGKQALE
TVQRLLPVLCQAHGLTPEQVVAIASNIGGKQALETVQRLLPVLCQAHGLTPEQVVAIASNI
GGKQALETVQRLLPVLCQAHGLTPEQVVAIASHDGGKQALETVQRLLPVLCQAHGLTPE
QVVAIASNGGGKQALETVQRLLPVLCQAHGLTPEQVVAIASNGGGKQALE**SIVAQLSRP**
DPALAALTNDHLVALACLGGRPALDAVKKGLGGSAVANNSRAIDYVKTYTGTSNDPKIS
PRYKGNSGGSTNLSDIIEKETGKQLVIQESILMLPEEVEEVIGNKPESDILVHTAYDESTDE
NVMLLTSDAPEYKPWALVIQDSNGENKIKMLSGVDGSGEGRGSLLTCGDVEENPGPPV
ATMVSKGEELFTGVVPILVELDGDVNGHKFSVSGEGEGDATYGKLTLKFICTTGKLPVPW
PTLVTTLTYGVQCFSRYPDHMKQHDFFKSAMPEGYVQERTIFFKDDGNYKTRAEVKFE
GDTLVNRIELKGIDFKEDGNILGHKLEYNYNSHNVYIMADKQKNGIKVNFKIRHNIEDGSV
QLADHYQQNTPIGDGPVLLPDNHYLSTQSALSKDPNEKRDHMVLLEFVTAAGITLGMDE
LYKLE

ATP6.1-DdCBE Left mitoTALE repeat
VDLRTLGYSQQQQEKIKPKVRSTVAQHHEALVGHGFTHAHIVALSQHPAALGTVAVKY
QDMIAALPEATHEAIVGVGKQWSGARALEALLTVAGELRGPPLQLDTGQLLKIAKRGG
VTAVEAVHAWRNALTGAPLNLTPEQVVAIASHDGGKQALETVQRLLPVLCQAHGLTPEQ
VVAIASHDGGKQALETVQRLLPVLCQAHGLTPEQVVAIASHDGGKQALETVQRLLPVLC
QAHGLTPEQVVAIASHDGGKQALETVQRLLPVLCQAHGLTPEQVVAIASNIGGKQALETV
QRLLPVLCQAHGLTPEQVVAIASNGGGKQALETVQRLLPVLCQAHGLTPEQVVAIASNIG
GKQALETVQRLLPVLCQAHGLTPEQVVAIASHDGGKQALETVQRLLPVLCQAHGLTPEQ
VVAIASNGGGKQALETVQRLLPVLCQAHGLTPEQVVAIASNIGGKQALETVQRLLPVLCQ
AHGLTPEQVVAIASNNGGKQALETVQRLLPVLCQAHGLTPEQVVAIASNGGGKQALETV
QRLLPVLCQAHGLTPEQVVAIASNGGGKQALETVQRLLPVLCQAHGLTPEQVVAIASNIG
GKQALETVQRLLPVLCQAHGLTPEQVVAIASNGGGKQALETVQRLLPVLCQAHGLTPEQ
VVAIASNGGGKQALE**SIVAQLSRPDPALAALTNDHLVALACLGGRPALDAVKKGLG**

ATP6.1-DdCBE Right mitoTALE repeat
VDLRTLGYSQQQQEKIKPKVRSTVAQHHEALVGHGFTHAHIVALSQHPAALGTVAVKY
QDMIAALPEATHEAIVGVGKQWSGARALEALLTVAGELRGPPLQLDTGQLLKIAKRGG
VTAVEAVHAWRNALTGAPLNLTPEQVVAIASNIGGKQALETVQRLLPVLCQAHGLTPEQ

VVAIASNGGGKQALETVQRLLPVLCQAHGLTPEQVVAIASNGGGKQALETVQRLLPVLC
QAHGLTPEQVVAIASNNGGKQALETVQRLLPVLCQAHGLTPEQVVAIASNNGGKQALET
VQRLLPVLCQAHGLTPEQVVAIASNGGGKQALETVQRLLPVLCQAHGLTPEQVVAIASN
GGGKQALETVQRLLPVLCQAHGLTPEQVVAIASNNGGKQALETVQRLLPVLCQAHGLTP
EQVVAIASNIGGKQALETVQRLLPVLCQAHGLTPEQVVAIASNIGGKQALETVQRLLPVLC
QAHGLTPEQVVAIASNGGGKQALETVQRLLPVLCQAHGLTPEQVVAIASNNGGKQALET
VQRLLPVLCQAHGLTPEQVVAIASNIGGKQALETVQRLLPVLCQAHGLTPEQVVAIASNN
GGKQALETVQRLLPVLCQAHGLTPEQVVAIASNGGGKQALE**SIVAQLSRPDPALAALTN
DHLVALACLGGRPALDAVKKGLG**

ND1-DdCBE Left mitoTALE repeat

**VDLRTLGYSQQQQEKIKPKVRSTVAQHHEALVGHGFTHAHIVALSQHPAALGTVAVKY
QDMIAALPEATHEAIVGVGKQWSGARALEALLTVAGELRGPPLQLDTGQLLKIAKRGG
VTAVEAVHAWRNALTGAPLN**LTPEQVVAIASHDGGKQALETVQRLLPVLCQAHGLTPEQ
VVAIASNIGGKQALETVQRLLPVLCQAHGLTPEQVVAIASNIGGKQALETVQRLLPVLCQA
HGLTPEQVVAIASNIGGKQALETVQRLLPVLCQAHGLTPEQVVAIASHDGGKQALETVQR
LLPVLCQAHGLTPEQVVAIASNGGGKQALETVQRLLPVLCQAHGLTPEQVVAIASNIGGK
QALETVQRLLPVLCQAHGLTPEQVVAIASHDGGKQALETVQRLLPVLCQAHGLTPEQVV
AIASNNGGKQALETVQRLLPVLCQAHGLTPEQVVAIASHDGGKQALETVQRLLPVLCQA
HGLTPEQVVAIASHDGGKQALETVQRLLPVLCQAHGLTPEQVVAIASHDGGKQALETVQ
RLLPVLCQAHGLTPEQVVAIASNGGGKQALE**SIVAQLSRPDPALAALTNDHLVALACLG
GRPALDAVKKGLG**

ND1-DdCBE Right mitoTALE repeat

**VDLRTLGYSQQQQEKIKPKVRSTVAQHHEALVGHGFTHAHIVALSQHPAALGTVAVKY
QDMIAALPEATHEAIVGVGKQWSGARALEALLTVAGELRGPPLQLDTGQLLKIAKRGG
VTAVEAVHAWRNALTGAPLN**LTPEQVVAIASNGGGKQALETVQRLLPVLCQAHGLTPEQ
VVAIASNNGGKQALETVQRLLPVLCQAHGLTPEQVVAIASNNGGKQALETVQRLLPVLC
QAHGLTPEQVVAIASNNGGKQALETVQRLLPVLCQAHGLTPEQVVAIASHDGGKQALET
VQRLLPVLCQAHGLTPEQVVAIASNGGGKQALETVQRLLPVLCQAHGLTPEQVVAIASNI
GGKQALETVQRLLPVLCQAHGLTPEQVVAIASHDGGKQALETVQRLLPVLCQAHGLTPE
QVVAIASNGGGKQALETVQRLLPVLCQAHGLTPEQVVAIASNNGGKQALETVQRLLPVL
CQAHGLTPEQVVAIASHDGGKQALETVQRLLPVLCQAHGLTPEQVVAIASNGGGKQALE
SIVAQLSRPDPALAALTNDHLVALACLGGRPALDAVKKGLG

TRNA-DdCBE Left mitoTALE repeat

**VDLRTLGYSQQQQEKIKPKVRSTVAQHHEALVGHGFTHAHIVALSQHPAALGTVAVKY
QDMIAALPEATHEAIVGVGKQWSGARALEALLTVAGELRGPPLQLDTGQLLKIAKRGG
VTAVEAVHAWRNALTGAPLN**LTPEQVVAIASNNGGKQALETVQRLLPVLCQAHGLTPEQ
VVAIASHDGGKQALETVQRLLPVLCQAHGLTPEQVVAIASNIGGKQALETVQRLLPVLCQ
AHGLTPEQVVAIASNIGGKQALETVQRLLPVLCQAHGLTPEQVVAIASNGGGKQALETVQ
RLLPVLCQAHGLTPEQVVAIASNIGGKQALETVQRLLPVLCQAHGLTPEQVVAIASHDGG

KQALETVQRLLPVLCQAHGLTPEQVVAIASNGGGKQALETVQRLLPVLCQAHGLTPEQV
VAIASNGGGKQALETVQRLLPVLCQAHGLTPEQVVAIASNIGGKQALETVQRLLPVLCQA
HGLTPEQVVAIASNIGGKQALETVQRLLPVLCQAHGLTPEQVVAIASNGGGKQALETVQR
LLPVLCQAHGLTPEQVVAIASNGGGKQALETVQRLLPVLCQAHGLTPEQVVAIASNGGG
KQALETVQRLLPVLCQAHGLTPEQVVAIASHDGGKQALETVQRLLPVLCQAHGLTPEQV
VAIASNGGGKQALE**SIVAQLSRPDPALAALTNDHLVALACLGGRPALDAVKKGLG**

TRNA-DdCBE Right mitoTALE repeat

**VDLRTLGYSQQQQEKIKPKVRSTVAQHHEALVGHGFTHAHIVALSQHPAALGTVAVKY
QDMIAALPEATHEAIVGVGKQWSGARALEALLTVAGELRGPPLQLDTGQLLKIAKRGG
VTAVEAVHAWRNALTGAPLN**LTPEQVVAIASNNGGKQALETVQRLLPVLCQAHGLTPEQ
VVAIASHDGGKQALETVQRLLPVLCQAHGLTPEQVVAIASNIGGKQALETVQRLLPVLCQ
AHGLTPEQVVAIASNNGGKQALETVQRLLPVLCQAHGLTPEQVVAIASNIGGKQALETVQ
RLLPVLCQAHGLTPEQVVAIASNNGGKQALETVQRLLPVLCQAHGLTPEQVVAIASNGG
GKQALETVQRLLPVLCQAHGLTPEQVVAIASNNGGKQALETVQRLLPVLCQAHGLTPEQ
VVAIASNNGGKQALETVQRLLPVLCQAHGLTPEQVVAIASNNGGKQALETVQRLLPVLC
QAHGLTPEQVVAIASNNGGKQALETVQRLLPVLCQAHGLTPEQVVAIASNGGGKQALET
VQRLLPVLCQAHGLTPEQVVAIASNGGGKQALETVQRLLPVLCQAHGLTPEQVVAIASN
GGGKQALETVQRLLPVLCQAHGLTPEQVVAIASNGGGKQALE**SIVAQLSRPDPALAALT
NDHLVALACLGGRPALDAVKKGLG**

ATP8-DdCBE Left mitoTALE repeat

**VDLRTLGYSQQQQEKIKPKVRSTVAQHHEALVGHGFTHAHIVALSQHPAALGTVAVKY
QDMIAALPEATHEAIVGVGKQWSGARALEALLTVAGELRGPPLQLDTGQLLKIAKRGG
VTAVEAVHAWRNALTGAPLN**LTPEQVVAIASNNGGKQALETVQRLLPVLCQAHGLTPEQ
VVAIASHDGGKQALETVQRLLPVLCQAHGLTPEQVVAIASHDGGKQALETVQRLLPVLC
QAHGLTPEQVVAIASHDGGKQALETVQRLLPVLCQAHGLTPEQVVAIASHDGGKQALET
VQRLLPVLCQAHGLTPEQVVAIASNIGGKQALETVQRLLPVLCQAHGLTPEQVVAIASNIG
GKQALETVQRLLPVLCQAHGLTPEQVVAIASHDGGKQALETVQRLLPVLCQAHGLTPEQ
VVAIASNGGGKQALETVQRLLPVLCQAHGLTPEQVVAIASNIGGKQALETVQRLLPVLCQ
AHGLTPEQVVAIASNIGGKQALETVQRLLPVLCQAHGLTPEQVVAIASNIGGKQALETVQ
RLLPVLCQAHGLTPEQVVAIASNGGGKQALETVQRLLPVLCQAHGLTPEQVVAIASNIGG
KQALETVQRLLPVLCQAHGLTPEQVVAIASHDGGKQALETVQRLLPVLCQAHGLTPEQV
VAIASNGGGKQALE**SIVAQLSRPDPALAALTNDHLVALACLGGRPALDAVKKGLG**

ATP8-DdCBE Right mitoTALE repeat

**VDLRTLGYSQQQQEKIKPKVRSTVAQHHEALVGHGFTHAHIVALSQHPAALGTVAVKY
QDMIAALPEATHEAIVGVGKQWSGARALEALLTVAGELRGPPLQLDTGQLLKIAKRGG
VTAVEAVHAWRNALTGAPLN**LTPEQVVAIASNIGGKQALETVQRLLPVLCQAHGLTPEQ
VVAIASNGGGKQALETVQRLLPVLCQAHGLTPEQVVAIASNNGGKQALETVQRLLPVLC
QAHGLTPEQVVAIASNNGGKQALETVQRLLPVLCQAHGLTPEQVVAIASNNGGKQALET
VQRLLPVLCQAHGLTPEQVVAIASNNGGKQALETVQRLLPVLCQAHGLTPEQVVAIASNN

GGKQALETVQRLLPVLCQAHGLTPEQVVAIASNGGGKQALETVQRLLPVLCQAHGLTPE
QVVAIASNIGGKQALETVQRLLPVLCQAHGLTPEQVVAIASNIGGKQALETVQRLLPVLC
QAHGLTPEQVVAIASNGGGKQALETVQRLLPVLCQAHGLTPEQVVAIASNGGGKQALET
VQRLLPVLCQAHGLTPEQVVAIASNIGGKQALETVQRLLPVLCQAHGLTPEQVVAIASNG
GGKQALE**SIVAQLSRPDPALAALTNDHLVALACLGGRPALDAVKKGLG**

ATP6.2-DdCBE Left mitoTALE repeat

VDLRTLGYSQQQQEKIKPKVRSTVAQHHEALVGHGFTHAHIVALSQHPAALGTVAVKY
QDMIAALPEATHEAIVGVGKQWSGARALEALLTVAGELRGPPLQLDTGQLLKIAKRGG
VTAVEAVHAWRNALTGAPLNLTPEQVVAIASNGGGKQALETVQRLLPVLCQAHGLTPEQ
VVAIASNNGGKQALETVQRLLPVLCQAHGLTPEQVVAIASHDGGKQALETVQRLLPVLC
QAHGLTPEQVVAIASHDGGKQALETVQRLLPVLCQAHGLTPEQVVAIASHDGGKQALET
VQRLLPVLCQAHGLTPEQVVAIASHDGGKQALETVQRLLPVLCQAHGLTPEQVVAIASHD
GGKQALETVQRLLPVLCQAHGLTPEQVVAIASNIGGKQALETVQRLLPVLCQAHGLTPE
QVVAIASHDGGKQALETVQRLLPVLCQAHGLTPEQVVAIASNIGGKQALETVQRLLPVLC
QAHGLTPEQVVAIASNIGGKQALETVQRLLPVLCQAHGLTPEQVVAIASNGGGKQALE**SI**
VAQLSRPDPALAALTNDHLVALACLGGRPALDAVKKGLG

ATP6.2-DdCBE Right mitoTALE repeat

VDLRTLGYSQQQQEKIKPKVRSTVAQHHEALVGHGFTHAHIVALSQHPAALGTVAVKY
QDMIAALPEATHEAIVGVGKQWSGARALEALLTVAGELRGPPLQLDTGQLLKIAKRGG
VTAVEAVHAWRNALTGAPLNLTPEQVVAIASNIGGKQALETVQRLLPVLCQAHGLTPEQ
VVAIASNNGGKQALETVQRLLPVLCQAHGLTPEQVVAIASNIGGKQALETVQRLLPVLCQ
AHGLTPEQVVAIASNIGGKQALETVQRLLPVLCQAHGLTPEQVVAIASNGGGKQALETVQ
RLLPVLCQAHGLTPEQVVAIASNNGGKQALETVQRLLPVLCQAHGLTPEQVVAIASNIGG
KQALETVQRLLPVLCQAHGLTPEQVVAIASNGGGKQALETVQRLLPVLCQAHGLTPEQV
VAIASHDGGKQALETVQRLLPVLCQAHGLTPEQVVAIASNIGGKQALETVQRLLPVLCQA
HGLTPEQVVAIASNNGGKQALETVQRLLPVLCQAHGLTPEQVVAIASNGGGKQALETVQ
RLLPVLCQAHGLTPEQVVAIASNIGGKQALETVQRLLPVLCQAHGLTPEQVVAIASHDGG
KQALETVQRLLPVLCQAHGLTPEQVVAIASNGGGKQALE**SIVAQLSRPDPALAALTNDH**
LVALACLGGRPALDAVKKGLG

ATP6.3-DdCBE Left mitoTALE repeat

VDLRTLGYSQQQQEKIKPKVRSTVAQHHEALVGHGFTHAHIVALSQHPAALGTVAVKY
QDMIAALPEATHEAIVGVGKQWSGARALEALLTVAGELRGPPLQLDTGQLLKIAKRGG
VTAVEAVHAWRNALTGAPLNLTPEQVVAIASHDGGKQALETVQRLLPVLCQAHGLTPEQ
VVAIASNIGGKQALETVQRLLPVLCQAHGLTPEQVVAIASNIGGKQALETVQRLLPVLCQA
HGLTPEQVVAIASHDGGKQALETVQRLLPVLCQAHGLTPEQVVAIASNIGGKQALETVQR
LLPVLCQAHGLTPEQVVAIASNIGGKQALETVQRLLPVLCQAHGLTPEQVVAIASHDGGK
QALETVQRLLPVLCQAHGLTPEQVVAIASHDGGKQALETVQRLLPVLCQAHGLTPEQVV
AIASNNGGKQALETVQRLLPVLCQAHGLTPEQVVAIASNIGGKQALETVQRLLPVLCQAH
GLTPEQVVAIASHDGGKQALETVQRLLPVLCQAHGLTPEQVVAIASNGGGKQALETVQR

LLPVLCQAHGLTPEQVVAIASNIGGKQALETVQRLLPVLCQAHGLTPEQVVAIASNIGGKQ
ALETVQRLLPVLCQAHGLTPEQVVAIASNGGGKQALE**SIVAQLSRPDPALAALTNDHLVA
LACLGGRPALDAVKKGLG**

ATP6.3-DdCBE Right mitoTALE repeat

**VDLRTLGYSQQQQEKIKPKVRSTVAQHHEALVGHGFTHAHIVALSQHPAALGTVAVKY
QDMIAALPEATHEAIVGVGKQWSGARALEALLTVAGELRGPPLQLDTGQLLKIAKRGG
VTAVEAVHAWRNALTGAPLN**LTPEQVVAIASNNGGKQALETVQRLLPVLCQAHGLTPEQ
VVAIASNIGGKQALETVQRLLPVLCQAHGLTPEQVVAIASNNGGKQALETVQRLLPVLCQ
AHGLTPEQVVAIASNNGGKQALETVQRLLPVLCQAHGLTPEQVVAIASNGGGKQALETV
QRLLPVLCQAHGLTPEQVVAIASNGGGKQALETVQRLLPVLCQAHGLTPEQVVAIASNIG
GKQALETVQRLLPVLCQAHGLTPEQVVAIASNNGGKQALETVQRLLPVLCQAHGLTPEQ
VVAIASNGGGKQALETVQRLLPVLCQAHGLTPEQVVAIASNGGGKQALETVQRLLPVLC
QAHGLTPEQVVAIASNGGGKQALETVQRLLPVLCQAHGLTPEQVVAIASNNGGKQALET
VQRLLPVLCQAHGLTPEQVVAIASNIGGKQALETVQRLLPVLCQAHGLTPEQVVAIASNG
GGKQALETVQRLLPVLCQAHGLTPEQVVAIASNGGGKQALE**SIVAQLSRPDPALAALTN
DHLVALACLGGRPALDAVKKGLG**

ATP6.4-DdCBE Left mitoTALE repeat

**VDLRTLGYSQQQQEKIKPKVRSTVAQHHEALVGHGFTHAHIVALSQHPAALGTVAVKY
QDMIAALPEATHEAIVGVGKQWSGARALEALLTVAGELRGPPLQLDTGQLLKIAKRGG
VTAVEAVHAWRNALTGAPLN**LTPEQVVAIASNNGGKQALETVQRLLPVLCQAHGLTPEQ
VVAIASNIGGKQALETVQRLLPVLCQAHGLTPEQVVAIASHDGGKQALETVQRLLPVLCQ
AHGLTPEQVVAIASNGGGKQALETVQRLLPVLCQAHGLTPEQVVAIASNIGGKQALETVQ
RLLPVLCQAHGLTPEQVVAIASNIGGKQALETVQRLLPVLCQAHGLTPEQVVAIASNGGG
KQALETVQRLLPVLCQAHGLTPEQVVAIASHDGGKQALETVQRLLPVLCQAHGLTPEQV
VAIASNIGGKQALETVQRLLPVLCQAHGLTPEQVVAIASNIGGKQALETVQRLLPVLCQAH
GLTPEQVVAIASNIGGKQALETVQRLLPVLCQAHGLTPEQVVAIASHDGGKQALETVQRL
LPVLCQAHGLTPEQVVAIASNGGGKQALE**SIVAQLSRPDPALAALTNDHLVALACLGGR
PALDAVKKGLG**

ATP6.4-DdCBE Right mitoTALE repeat

**VDLRTLGYSQQQQEKIKPKVRSTVAQHHEALVGHGFTHAHIVALSQHPAALGTVAVKY
QDMIAALPEATHEAIVGVGKQWSGARALEALLTVAGELRGPPLQLDTGQLLKIAKRGG
VTAVEAVHAWRNALTGAPLN**LTPEQVVAIASNIGGKQALETVQRLLPVLCQAHGLTPEQ
VVAIASNNGGKQALETVQRLLPVLCQAHGLTPEQVVAIASNGGGKQALETVQRLLPVLC
QAHGLTPEQVVAIASNNGGKQALETVQRLLPVLCQAHGLTPEQVVAIASNGGGKQALET
VQRLLPVLCQAHGLTPEQVVAIASNGGGKQALETVQRLLPVLCQAHGLTPEQVVAIASN
NGGKQALETVQRLLPVLCQAHGLTPEQVVAIASNGGGKQALETVQRLLPVLCQAHGLTP
EQVVAIASNNGGKQALETVQRLLPVLCQAHGLTPEQVVAIASNGGGKQALETVQRLLPV
LCQAHGLTPEQVVAIASNIGGKQALETVQRLLPVLCQAHGLTPEQVVAIASNGGGKQALE
TVQRLLPVLCQAHGLTPEQVVAIASNNGGKQALETVQRLLPVLCQAHGLTPEQVVAIASN

NGGKQALETVQRLLPVLCQAHGLTPEQVVAIASNGGGKQALETVQRLLPVLCQAHGLTPEQVVAIASNGGGKQALE**SIVAQLSRPDPALAALTNDHLVALACLGGRPALDAVKKGLG**

COX3-DdCBE Left mitoTALE repeat

VDLRTLGYSQQQQEKIKPKVRSTVAQHHEALVGHGFTHAHIVALSQHPAALGTVAVKYQDMIAALPEATHEAIVGVGKQWSGARALEALLTVAGELRGPPLQLDTGQLLKIAKRGGVTAVEAVHAWRNALTGAPLNLTPEQVVAIASHDGGKQALETVQRLLPVLCQAHGLTPEQVVAIASNNGGKQALETVQRLLPVLCQAHGLTPEQVVAIASNIGGKQALETVQRLLPVLCQAHGLTPEQVVAIASNIGGKQALETVQRLLPVLCQAHGLTPEQVVAIASNNGGKQALETVQRLLPVLCQAHGLTPEQVVAIASHDGGKQALETVQRLLPVLCQAHGLTPEQVVAIASHDGGKQALETVQRLLPVLCQAHGLTPEQVVAIASNNGGKQALETVQRLLPVLCQAHGLTPEQVVAIASHDGGKQALETVQRLLPVLCQAHGLTPEQVVAIASHDGGKQALETVQRLLPVLCQAHGLTPEQVVAIASNNGGKQALETVQRLLPVLCQAHGLTPEQVVAIASHDGGKQALETVQRLLPVLCQAHGLTPEQVVAIASHDGGKQALETVQRLLPVLCQAHGLTPEQVVAIASNGGGKQALE**SIVAQLSRPDPALAALTNDHLVALACLGGRPALDAVKKGLG**

COX3-DdCBE Right mitoTALE repeat

VDLRTLGYSQQQQEKIKPKVRSTVAQHHEALVGHGFTHAHIVALSQHPAALGTVAVKYQDMIAALPEATHEAIVGVGKQWSGARALEALLTVAGELRGPPLQLDTGQLLKIAKRGGVTAVEAVHAWRNALTGAPLNLTPEQVVAIASNIGGKQALETVQRLLPVLCQAHGLTPEQVVAIASNNGGKQALETVQRLLPVLCQAHGLTPEQVVAIASNGGGKQALETVQRLLPVLCQAHGLTPEQVVAIASHDGGKQALETVQRLLPVLCQAHGLTPEQVVAIASNIGGKQALETVQRLLPVLCQAHGLTPEQVVAIASNIGGKQALETVQRLLPVLCQAHGLTPEQVVAIASNIGGKQALETVQRLLPVLCQAHGLTPEQVVAIASHDGGKQALETVQRLLPVLCQAHGLTPEQVVAIASHDGGKQALETVQRLLPVLCQAHGLTPEQVVAIASNIGGKQALETVQRLLPVLCQAHGLTPEQVVAIASHDGGKQALETVQRLLPVLCQAHGLTPEQVVAIASNIGGKQALETVQRLLPVLCQAHGLTPEQVVAIASNGGGKQALETVQRLLPVLCQAHGLTPEQVVAIASHDGGKQALETVQRLLPVLCQAHGLTPEQVVAIASNGGGKQALE**SIVAQLSRPDPALAALTNDHLVALACLGGRPALDAVKKGLG**

ND4-DdCBE Left mitoTALE repeat

VDLRTLGYSQQQQEKIKPKVRSTVAQHHEALVGHGFTHAHIVALSQHPAALGTVAVKYQDMIAALPEATHEAIVGVGKQWSGARALEALLTVAGELRGPPLQLDTGQLLKIAKRGGVTAVEAVHAWRNALTGAPLNLTPEQVVAIASNIGGKQALETVQRLLPVLCQAHGLTPEQVVAIASHDGGKQALETVQRLLPVLCQAHGLTPEQVVAIASNNGGKQALETVQRLLPVLCQAHGLTPEQVVAIASNIGGKQALETVQRLLPVLCQAHGLTPEQVVAIASNIGGKQALETVQRLLPVLCQAHGLTPEQVVAIASHDGGKQALETVQRLLPVLCQAHGLTPEQVVAIASNNGGKQALETVQRLLPVLCQAHGLTPEQVVAIASHDGGKQALETVQRLLPVLCQAHGLTPEQVVAIASNIGGKQALETVQRLLPVLCQAHGLTPEQVVAIASHDGGKQALETVQRLLPVLCQAHGLTPEQVVAIASNGGGKQALE**SIVAQLSRPDPALAALTNDHLVALACLGGRPALDAVKKGLG**

ND4-DdCBE Right mitoTALE repeat

VDLRTLGYSQQQQEKIKPKVRSTVAQHHEALVGHGFTHAHIVALSQHPAALGTVAVKY
QDMIAALPEATHEAIVGVGKQWSGARALEALLTVAGELRGPPLQLDTGQLLKIAKRGG
VTAVEAVHAWRNALTGAPLNLTPEQVVAIASHDGGKQALETVQRLLPVLCQAHGLTPEQ
VVAIASHDGGKQALETVQRLLPVLCQAHGLTPEQVVAIASNGGGKQALETVQRLLPVLC
QAHGLTPEQVVAIASNGGGKQALETVQRLLPVLCQAHGLTPEQVVAIASNNGGKQALET
VQRLLPVLCQAHGLTPEQVVAIASNIGGKQALETVQRLLPVLCQAHGLTPEQVVAIASNN
GGKQALETVQRLLPVLCQAHGLTPEQVVAIASNIGGKQALETVQRLLPVLCQAHGLTPE
QVVAIASNNGGKQALETVQRLLPVLCQAHGLTPEQVVAIASNIGGKQALETVQRLLPVLC
QAHGLTPEQVVAIASNNGGKQALETVQRLLPVLCQAHGLTPEQVVAIASNNGGKQALET
VQRLLPVLCQAHGLTPEQVVAIASNIGGKQALETVQRLLPVLCQAHGLTPEQVVAIASNG
GGKQALETVQRLLPVLCQAHGLTPEQVVAIASNGGGKQALE**SIVAQLSRPDPALAALTN**
DHLVALACLGGRPALDAVKKGLG

TRNS2-DdCBE Left mitoTALE repeat

VDLRTLGYSQQQQEKIKPKVRSTVAQHHEALVGHGFTHAHIVALSQHPAALGTVAVKY
QDMIAALPEATHEAIVGVGKQWSGARALEALLTVAGELRGPPLQLDTGQLLKIAKRGG
VTAVEAVHAWRNALTGAPLNLTPEQVVAIASNIGGKQALETVQRLLPVLCQAHGLTPEQ
VVAIASHDGGKQALETVQRLLPVLCQAHGLTPEQVVAIASHDGGKQALETVQRLLPVLC
QAHGLTPEQVVAIASNNGGKQALETVQRLLPVLCQAHGLTPEQVVAIASNIGGKQALETV
QRLLPVLCQAHGLTPEQVVAIASNNGGKQALETVQRLLPVLCQAHGLTPEQVVAIASNIG
GKQALETVQRLLPVLCQAHGLTPEQVVAIASNIGGKQALETVQRLLPVLCQAHGLTPEQV
VAIASNIGGKQALETVQRLLPVLCQAHGLTPEQVVAIASNNGGKQALETVQRLLPVLCQA
HGLTPEQVVAIASHDGGKQALETVQRLLPVLCQAHGLTPEQVVAIASNGGGKQALE**SIVA**
QLSRPDPALAALTNDHLVALACLGGRPALDAVKKGLG

TRNS2-DdCBE Right mitoTALE repeat

VDLRTLGYSQQQQEKIKPKVRSTVAQHHEALVGHGFTHAHIVALSQHPAALGTVAVKY
QDMIAALPEATHEAIVGVGKQWSGARALEALLTVAGELRGPPLQLDTGQLLKIAKRGG
VTAVEAVHAWRNALTGAPLNLTPEQVVAIASNIGGKQALETVQRLLPVLCQAHGLTPEQ
VVAIASNNGGKQALETVQRLLPVLCQAHGLTPEQVVAIASNIGGKQALETVQRLLPVLCQ
AHGLTPEQVVAIASHDGGKQALETVQRLLPVLCQAHGLTPEQVVAIASNIGGKQALETVQ
RLLPVLCQAHGLTPEQVVAIASNGGGKQALETVQRLLPVLCQAHGLTPEQVVAIASNNG
GKQALETVQRLLPVLCQAHGLTPEQVVAIASNNGGKQALETVQRLLPVLCQAHGLTPEQ
VVAIASNNGGKQALETVQRLLPVLCQAHGLTPEQVVAIASNNGGKQALETVQRLLPVLC
QAHGLTPEQVVAIASNNGGKQALETVQRLLPVLCQAHGLTPEQVVAIASHDGGKQALET
VQRLLPVLCQAHGLTPEQVVAIASNIGGKQALETVQRLLPVLCQAHGLTPEQVVAIASNG
GGKQALE**SIVAQLSRPDPALAALTNDHLVALACLGGRPALDAVKKGLG**

ND5.2-DdCBE Left mitoTALE repeat

VDLRTLGYSQQQQEKIKPKVRSTVAQHHEALVGHGFTHAHIVALSQHPAALGTVAVKY
QDMIAALPEATHEAIVGVGKQWSGARALEALLTVAGELRGPPLQLDTGQLLKIAKRGG

VTAVEAVHAWRNALTGAPLNLTPEQVVAIASHDGGKQALETVQRLLPVLCQAHGLTPEQ
VVAIASHDGGKQALETVQRLLPVLCQAHGLTPEQVVAIASHDGGKQALETVQRLLPVLC
QAHGLTPEQVVAIASHDGGKQALETVQRLLPVLCQAHGLTPEQVVAIASNGGGKQALET
VQRLLPVLCQAHGLTPEQVVAIASHDGGKQALETVQRLLPVLCQAHGLTPEQVVAIASNI
GGKQALETVQRLLPVLCQAHGLTPEQVVAIASNNGGKQALETVQRLLPVLCQAHGLTPE
QVVAIASHDGGKQALETVQRLLPVLCQAHGLTPEQVVAIASHDGGKQALETVQRLLPVL
CQAHGLTPEQVVAIASNIGGKQALETVQRLLPVLCQAHGLTPEQVVAIASNGGGKQALE**S**
IVAQLSRPDPALAALTNDHLVALACLGGRPALDAVKKGLG

ND5.2-DdCBE Right mitoTALE repeat

VDLRTLGYSQQQQEKIKPKVRSTVAQHHEALVGHGFTHAHIVALSQHPAALGTVAVKY
QDMIAALPEATHEAIVGVGKQWSGARALEALLTVAGELRGPPLQLDTGQLLKIAKRGG
VTAVEAVHAWRNALTGAPLNLTPEQVVAIASNIGGKQALETVQRLLPVLCQAHGLTPEQ
VVAIASNNGGKQALETVQRLLPVLCQAHGLTPEQVVAIASNNGGKQALETVQRLLPVLC
QAHGLTPEQVVAIASNNGGKQALETVQRLLPVLCQAHGLTPEQVVAIASHDGGKQALET
VQRLLPVLCQAHGLTPEQVVAIASNGGGKQALETVQRLLPVLCQAHGLTPEQVVAIASN
NGGKQALETVQRLLPVLCQAHGLTPEQVVAIASNIGGKQALETVQRLLPVLCQAHGLTP
EQVVAIASNNGGKQALETVQRLLPVLCQAHGLTPEQVVAIASNIGGKQALETVQRLLPVL
CQAHGLTPEQVVAIASHDGGKQALETVQRLLPVLCQAHGLTPEQVVAIASNGGGKQALE
SIVAQLSRPDPALAALTNDHLVALACLGGRPALDAVKKGLG

ND6-DdCBE Left mitoTALE repeat

VDLRTLGYSQQQQEKIKPKVRSTVAQHHEALVGHGFTHAHIVALSQHPAALGTVAVKY
QDMIAALPEATHEAIVGVGKQWSGARALEALLTVAGELRGPPLQLDTGQLLKIAKRGG
VTAVEAVHAWRNALTGAPLNLTPEQVVAIASHDGGKQALETVQRLLPVLCQAHGLTPEQ
VVAIASNIGGKQALETVQRLLPVLCQAHGLTPEQVVAIASNNGGKQALETVQRLLPVLCQ
AHGLTPEQVVAIASNNGGKQALETVQRLLPVLCQAHGLTPEQVVAIASNIGGKQALETVQ
RLLPVLCQAHGLTPEQVVAIASNGGGKQALETVQRLLPVLCQAHGLTPEQVVAIASNIGG
KQALETVQRLLPVLCQAHGLTPEQVVAIASHDGGKQALETVQRLLPVLCQAHGLTPEQV
VAIASNGGGKQALETVQRLLPVLCQAHGLTPEQVVAIASHDGGKQALETVQRLLPVLCQ
AHGLTPEQVVAIASHDGGKQALETVQRLLPVLCQAHGLTPEQVVAIASNGGGKQALETV
QRLLPVLCQAHGLTPEQVVAIASHDGGKQALETVQRLLPVLCQAHGLTPEQVVAIASNIG
GKQALETVQRLLPVLCQAHGLTPEQVVAIASNIGGKQALETVQRLLPVLCQAHGLTPEQV
VAIASNGGGKQALE**SIVAQLSRPDPALAALTNDHLVALACLGGRPALDAVKKGLG**

ND6-DdCBE Right mitoTALE repeat

VDLRTLGYSQQQQEKIKPKVRSTVAQHHEALVGHGFTHAHIVALSQHPAALGTVAVKY
QDMIAALPEATHEAIVGVGKQWSGARALEALLTVAGELRGPPLQLDTGQLLKIAKRGG
VTAVEAVHAWRNALTGAPLNLTPEQVVAIASNNGGKQALETVQRLLPVLCQAHGLTPEQ
VVAIASNNGGKQALETVQRLLPVLCQAHGLTPEQVVAIASNGGGKQALETVQRLLPVLC
QAHGLTPEQVVAIASNGGGKQALETVQRLLPVLCQAHGLTPEQVVAIASNNGGKQALET
VQRLLPVLCQAHGLTPEQVVAIASNGGGKQALETVQRLLPVLCQAHGLTPEQVVAIASH

DGGKQALETVQRLLPVLCQAHGLTPEQVVAIASNGGGKQALETVQRLLPVLCQAHGLTP
EQVVAIASNGGGKQALETVQRLLPVLCQAHGLTPEQVVAIASNGGGKQALETVQRLLPV
LCQAHGLTPEQVVAIASNNGGKQALETVQRLLPVLCQAHGLTPEQVVAIASNNGGKQAL
ETVQRLLPVLCQAHGLTPEQVVAIASNIGGKQALETVQRLLPVLCQAHGLTPEQVVAIAS
NGGGKQALE**SIVAQLSRPDPALAALTNDHLVALACLGGRPALDAVKKGLG**

CYTB-DdCBE Left mitoTALE repeat

**VDLRTLGYSQQQQEKIKPKVRSTVAQHHEALVGHGFTHAHIVALSQHPAALGTVAVKY
QDMIAALPEATHEAIVGVGKQWSGARALEALLTVAGELRGPPLQLDTGQLLKIAKRGG
VTAVEAVHAWRNALTGAPLN**LTPEQVVAIASHDGGKQALETVQRLLPVLCQAHGLTPEQ
VVAIASNIGGKQALETVQRLLPVLCQAHGLTPEQVVAIASHDGGKQALETVQRLLPVLCQ
AHGLTPEQVVAIASNGGGKQALETVQRLLPVLCQAHGLTPEQVVAIASHDGGKQALETV
QRLLPVLCQAHGLTPEQVVAIASNNGGKQALETVQRLLPVLCQAHGLTPEQVVAIASNIG
GKQALETVQRLLPVLCQAHGLTPEQVVAIASNNGGKQALETVQRLLPVLCQAHGLTPEQ
VVAIASNIGGKQALETVQRLLPVLCQAHGLTPEQVVAIASHDGGKQALETVQRLLPVLCQ
AHGLTPEQVVAIASNNGGKQALETVQRLLPVLCQAHGLTPEQVVAIASNGGGKQALE**SIV
AQLSRPDPALAALTNDHLVALACLGGRPALDAVKKGLG**

CYTB-DdCBE Right mitoTALE repeat

**VDLRTLGYSQQQQEKIKPKVRSTVAQHHEALVGHGFTHAHIVALSQHPAALGTVAVKY
QDMIAALPEATHEAIVGVGKQWSGARALEALLTVAGELRGPPLQLDTGQLLKIAKRGG
VTAVEAVHAWRNALTGAPLN**LTPEQVVAIASNNGGKQALETVQRLLPVLCQAHGLTPEQ
VVAIASNIGGKQALETVQRLLPVLCQAHGLTPEQVVAIASNIGGKQALETVQRLLPVLCQA
HGLTPEQVVAIASNNGGKQALETVQRLLPVLCQAHGLTPEQVVAIASNNGGKQALETVQ
RLLPVLCQAHGLTPEQVVAIASNGGGKQALETVQRLLPVLCQAHGLTPEQVVAIASNIGG
KQALETVQRLLPVLCQAHGLTPEQVVAIASNNGGKQALETVQRLLPVLCQAHGLTPEQV
VAIASHDGGKQALETVQRLLPVLCQAHGLTPEQVVAIASNNGGKQALETVQRLLPVLCQ
AHGLTPEQVVAIASNNGGKQALETVQRLLPVLCQAHGLTPEQVVAIASNIGGKQALETVQ
RLLPVLCQAHGLTPEQVVAIASNGGGKQALE**SIVAQLSRPDPALAALTNDHLVALACLG
GRPALDAVKKGLG**

Supplementary Note 2 | Ddd_Bc and Ddd_Ss variants used to construct DdCBEs.

Ddd_Ss1 (T26I+T77I+T110I)
SLPEYDGTTTHGVLVLDDGTQIGFISGNGDPRYTNYRNNGHVEQKSALYMRENNISNAT
VYHNNTNGTCGYCNTMIATFLPEGATLTVVPPENAVANNSRAIDYVKTYIGTSNDPKISPR
YKGN
Ddd_Ss2 (T26I)
SLPEYDGTTTHGVLVLDDGTQIGFISGNGDPRYTNYRNNGHVEQKSALYMRENNISNAT
VYHNNTNGTCGYCNTMTATFLPEGATLTVVPPENAVANNSRAIDYVKTYTGTSNDPKISP
RYKGN
Ddd_Ss3 (T77I)
SLPEYDGTTTHGVLVLDDGTQIGFTSGNGDPRYTNYRNNGHVEQKSALYMRENNISNAT
VYHNNTNGTCGYCNTMIATFLPEGATLTVVPPENAVANNSRAIDYVKTYTGTSNDPKISP
RYKGN
Ddd_Ss4 (T110I)
SLPEYDGTTTHGVLVLDDGTQIGFTSGNGDPRYTNYRNNGHVEQKSALYMRENNISNAT
VYHNNTNGTCGYCNTMTATFLPEGATLTVVPPENAVANNSRAIDYVKTYIGTSNDPKISP
RYKGN
Ddd_Ss5 (T77I+T110I)
SLPEYDGTTTHGVLVLDDGTQIGFTSGNGDPRYTNYRNNGHVEQKSALYMRENNISNAT
VYHNNTNGTCGYCNTMIATFLPEGATLTVVPPENAVANNSRAIDYVKTYIGTSNDPKISPR
YKGN
Ddd_Bc_L1
GSYALGPYQISAPQLPAYNGQTVGTFYYVNDAGGLESKVFSSGNGDPRYTNYRNNGHV
EGQSALFMRDNGISEGLVFHNNPEGTCGFCVNMTETLLPENAKMTVVPPEGAIPVKRG
ATGETKVFTGNSNSPKSPTKGGC
Ddd_Bc_P1369T
GSYALGPYQISAPQLPAYNGQTVGTFYYVNDAGGLESKVFSSGGPTPYPNYANAGHVE
GQSALFMRDNGISEGLVFHNNTEGTCGFCVNMTETLLPENAKMTVVPPEGAIPVKRGAT
GETKVFTGNSNSPKSPTKGGC
Ddd_Bc_E1370N
GSYALGPYQISAPQLPAYNGQTVGTFYYVNDAGGLESKVFSSGGPTPYPNYANAGHVE
GQSALFMRDNGISEGLVFHNNPNGTCGFCVNMTETLLPENAKMTVVPPEGAIPVKRGA
TGETKVFTGNSNSPKSPTKGGC
Ddd_Bc_L3
GSYALGPYQISAPQLPAYNGQTVGTFYYVNDAGGLESKVFSSGGPTPYPNYANAGHVE
GQSALFMRDNGISEGLVFHNNPEGTCGFCVNMTETLLPENAKMTVVPPEGAVANNSRAI
DYVKVFTGNSNSPKSPTKGGC

Supplementary Note 3 | Flow cytometric pseudo-color plots showing gating strategy. The cells were initially gated on population using SSC-A/FSC-A (Gate A), then sorted for single cells using FSC-W/FSC-H (Gate Single Cell). Double positive live cells were sorted by gating mCherry-positive and GFP-positive cells (Gate Double Positive). Untreated HEK293T cells were employed as negative control for generating gate Double Positive.

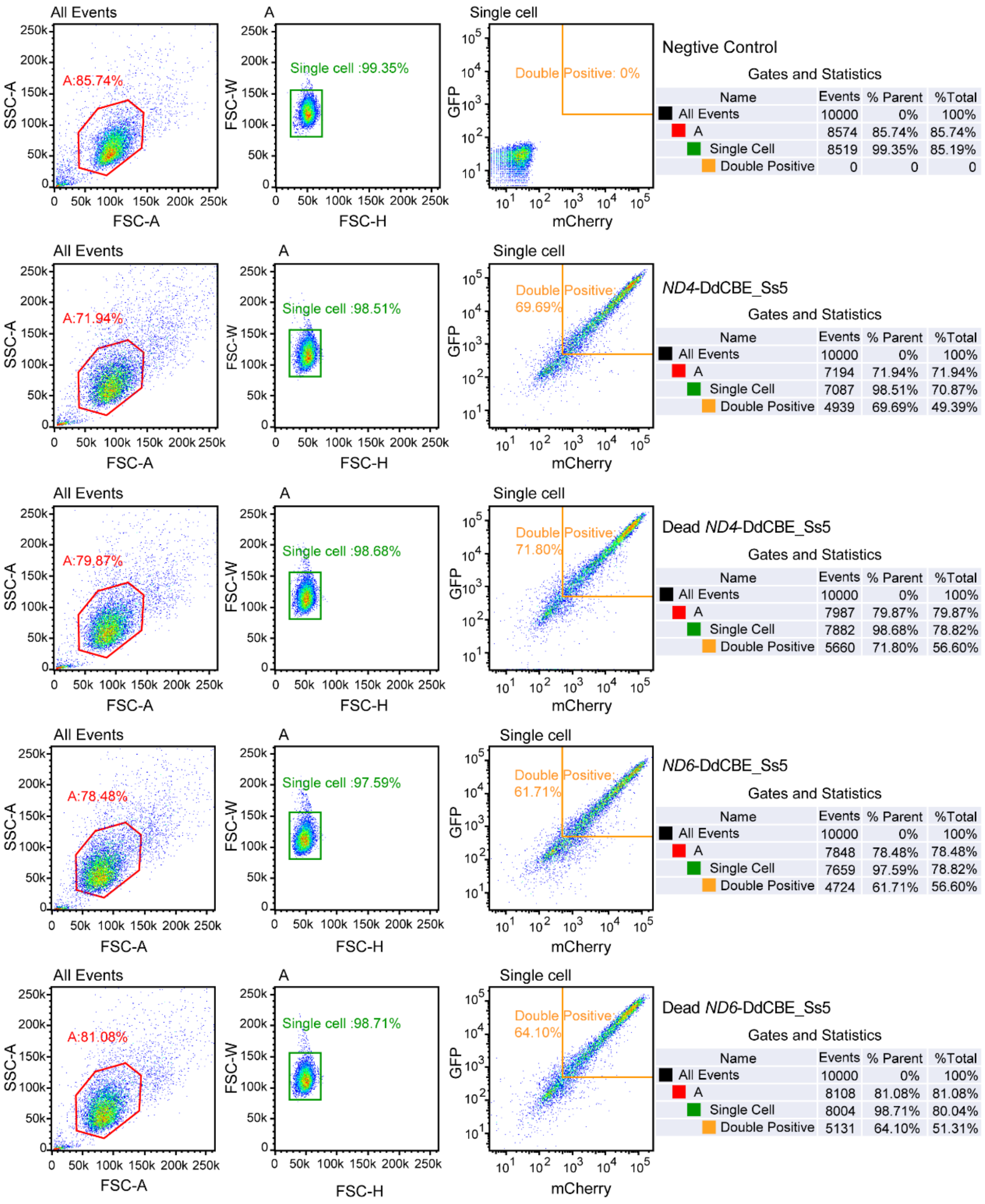

Supplement: Supplementary file 1 — Supplementary Information [file 41467_2023_36600_MOESM1_ESM.pdf]
