# Supplementary material for: DddA homolog search and engineering expand sequence compatibility of mitochondrial base editing
Source: Nat Commun. 2023 Feb 16;14:874. doi: 10.1038/s41467-023-36600-2 (PMC9935910; doi:10.1038/s41467-023-36600-2)
Supplement: Supplementary file 2 — Reporting Summary [file 41467_2023_36600_MOESM2_ESM.pdf]

nature portfolio

Corresponding author(s): Dr. Yangming Wang

Last updated by author(s): Feb 9, 2023

Reporting Summary

Nature Portfolio wishes to improve the reproducibility of the work that we publish. This form provides structure for consistency and transparency in reporting. For further information on Nature Portfolio policies, see our Editorial Policies and the Editorial Policy Checklist.

Statistics

For all statistical analyses, confirm that the following items are present in the figure legend, table legend, main text, or Methods section.

n/a	Confirmed	
☐	☒	The exact sample size (*n*) for each experimental group/condition, given as a discrete number and unit of measurement
☐	☒	A statement on whether measurements were taken from distinct samples or whether the same sample was measured repeatedly
☐	☒	The statistical test(s) used AND whether they are one- or two-sided<br>*Only common tests should be described solely by name; describe more complex techniques in the Methods section.*
☒	☐	A description of all covariates tested
☒	☐	A description of any assumptions or corrections, such as tests of normality and adjustment for multiple comparisons
☐	☒	A full description of the statistical parameters including central tendency (e.g. means) or other basic estimates (e.g. regression coefficient) AND variation (e.g. standard deviation) or associated estimates of uncertainty (e.g. confidence intervals)
☐	☒	For null hypothesis testing, the test statistic (e.g. *F*, *t*, *r*) with confidence intervals, effect sizes, degrees of freedom and *P* value noted<br>*Give P values as exact values whenever suitable.*
☒	☐	For Bayesian analysis, information on the choice of priors and Markov chain Monte Carlo settings
☒	☐	For hierarchical and complex designs, identification of the appropriate level for tests and full reporting of outcomes
☒	☐	Estimates of effect sizes (e.g. Cohen's *d*, Pearson's *r*), indicating how they were calculated

Our web collection on statistics for biologists contains articles on many of the points above.

Software and code

Policy information about availability of computer code

Data collection	High throughput deep sequencing data were collected on Illumina Nova-seq 6000 and MGISEQ-2000.
Data analysis	Bacterial genome SNVs were called using VarScan2 (version 2.4.4). High-throughput sequencing data of targeted amplicon was analyzed using CRISPResso2 (version 2.2.7). mtDNA off-target effects from ATAC-seq data were calculated using REDItools (version 1.2.1). Detect-seq data were processed using Detect-seq tool (www.detect-seq.com). For data analysis and visualization, we used GraphPad Prism 6, BWA software (version 0.7.17), Picard tools (version 2.18.29), SAMtools (version 1.14), WebLogo 3 online tool (version 3.7.12, https://weblogo.threeplusone.com), Microsoft Excel 2019, and Adobe Illustrator CC2018.

For manuscripts utilizing custom algorithms or software that are central to the research but not yet described in published literature, software must be made available to editors and reviewers. We strongly encourage code deposition in a community repository (e.g. GitHub). See the Nature Portfolio guidelines for submitting code & software for further information.

Data

Policy information about availability of data

All manuscripts must include a data availability statement. This statement should provide the following information, where applicable:

- Accession codes, unique identifiers, or web links for publicly available datasets
- A description of any restrictions on data availability
- For clinical datasets or third party data, please ensure that the statement adheres to our policy

The high-throughput sequencing data generated in this study have been deposited in the NCBI's Sequence Read Archive (SRA) database under accession code

PRJNA915236 (https://www.ncbi.nlm.nih.gov/bioproject/PRJNA915236). NC_000913.3 (https://www.ncbi.nlm.nih.gov/nuccore/NC_000913.3) was used as E.coli reference genome. NC_012920.1 (https://www.ncbi.nlm.nih.gov/nuccore/NC_012920.1) was used as human mitochondrial reference genome. Amino acid sequences to construct DdCBEs in this study are provided in Supplementary Notes 1 and 2. Source data are provided with this paper.

Human research participants

Policy information about studies involving human research participants and Sex and Gender in Research.

Reporting on sex and gender	N/A
Population characteristics	N/A
Recruitment	N/A
Ethics oversight	N/A

Note that full information on the approval of the study protocol must also be provided in the manuscript.

Field-specific reporting

Please select the one below that is the best fit for your research. If you are not sure, read the appropriate sections before making your selection.

[x] Life sciences [ ] Behavioural & social sciences [ ] Ecological, evolutionary & environmental sciences

For a reference copy of the document with all sections, see nature.com/documents/nr-reporting-summary-flat.pdf

Life sciences study design

All studies must disclose on these points even when the disclosure is negative.

Sample size	No statistical analysis were performed to predetermine sample size. Sample sizes were chosen on the basis of existing procedures and standards in the field. For all experiments, at least 2 (n>=2) independent biological replicates were performed. Our results show that it is sufficient to yield reproducible mean results values. So the sample size we used is sufficient to support conclusions in this paper.
Data exclusions	No data were excluded from the analyses.
Replication	All experiments were performed from individual biological replicates (n=2 or 3). All attempts at replication were successful.
Randomization	All independent biological replicates were treated identically. Thus randomization was not relevant to this study.
Blinding	All independent biological replicates were treated identically without preference, so blinding was not used.

Reporting for specific materials, systems and methods

We require information from authors about some types of materials, experimental systems and methods used in many studies. Here, indicate whether each material, system or method listed is relevant to your study. If you are not sure if a list item applies to your research, read the appropriate section before selecting a response.

Materials & experimental systems

n/a	Involved in the study	
[x]	[ ]	Antibodies
[ ]	[x]	Eukaryotic cell lines
[x]	[ ]	Palaeontology and archaeology
[x]	[ ]	Animals and other organisms
[x]	[ ]	Clinical data
[x]	[ ]	Dual use research of concern

Methods

n/a	Involved in the study	
[x]	[ ]	ChIP-seq
[ ]	[x]	Flow cytometry
[x]	[ ]	MRI-based neuroimaging

Eukaryotic cell lines

Policy information about cell lines and Sex and Gender in Research

Cell line source(s)	HEK293T (ATCC, CRL-3216), HeLa (ATCC, CCL-2) and U2OS (ATCC, HTB-96)
Authentication	The cell line was not authenticated.
Mycoplasma contamination	The cell line was not tested for mycoplasma contamination.
Commonly misidentified lines (See ICLAC register)	No commonly misidentified lines were used.

Flow Cytometry

Plots

Confirm that:

- [x] The axis labels state the marker and fluorochrome used (e.g. CD4-FITC).
- [x] The axis scales are clearly visible. Include numbers along axes only for bottom left plot of group (a 'group' is an analysis of identical markers).
- [x] All plots are contour plots with outliers or pseudocolor plots.
- [x] A numerical value for number of cells or percentage (with statistics) is provided.

Methodology

Sample preparation	HEK293T cells were transfected with 250ng of each DdCBE monomer as described in the methods, 3 days following transfection, cells were digested and resuspended in ice-cold culture medium. Cell suspensions were filtered through 40 micrometer cell strainers to remove debris.
Instrument	BD FACSAria III
Software	BD FACSDiva Software v8.0, FlowJo v10
Cell population abundance	The abundance of GFP+/mCherry+ cells were the ratio of double positive cell number to total cell number.The sorted double positive cells were collected in cell culture medium, and the density of collected cells were 200,000/mL.
Gating strategy	The cells were initially gated on population using SSC-A/FSC-A, then sorted for single cells using FSC-W/FSC-H. Untreated HEK293T cells were employed as negative control for generating gate Double Positive.

- [x] Tick this box to confirm that a figure exemplifying the gating strategy is provided in the Supplementary Information.
