# Supplementary material for: DddA homolog search and engineering expand sequence compatibility of mitochondrial base editing
Source: Nat Commun. 2023 Feb 16;14:874. doi: 10.1038/s41467-023-36600-2 (PMC9935910; doi:10.1038/s41467-023-36600-2)
Supplement: Supplementary file 6 — Description of Additional Supplementary Files [file 41467_2023_36600_MOESM6_ESM.pdf]

Title: Supplementary Data 1
Description: Candidate Ddd_Bc homologs by PSI-BLAST.

Title: Supplementary Data 2
Description: MitoTALE binding sites and editing efficiency for DdCBE.
